# Near‐infrared‐induced drug release from antibody–drug double conjugates exerts a cytotoxic photo‐bystander effect

**DOI:** 10.1002/btm2.10388

**Published:** 2022-08-21

**Authors:** Kazuomi Takahashi, Hirotoshi Yasui, Shunichi Taki, Misae Shimizu, Chiaki Koike, Kentaro Taki, Hiroshi Yukawa, Yoshinobu Baba, Hisataka Kobayashi, Kazuhide Sato

**Affiliations:** ^1^ Department of Respiratory Medicine Nagoya University Graduate School of Medicine Showa‐ku Nagoya Japan; ^2^ Nagoya University Institute for Advanced Research Advanced Analytical and Diagnostic Imaging Center (AADIC)/Medical Engineering Unit (MEU), B3 Unit Showa‐ku Nagoya Japan; ^3^ Division for Medical Research Engineering Nagoya University Graduate School of Medicine Showa‐ku Nagoya Japan; ^4^ Institute of Nano‐Life‐Systems, Institutes of Innovation for Future Society Nagoya University Nagoya Japan; ^5^ Department of Biomolecular Engineering Nagoya University Graduate School of Engineering Nagoya Japan; ^6^ Molecular Imaging Program National Cancer Institute, National Institutes of Health Bethesda Maryland USA; ^7^ FOREST‐Souhatsu, CREST, JST Chiyoda‐ku Tokyo Japan; ^8^ Nagoya University Institute for Advanced Research, S‐YLC Japan

**Keywords:** antibody–drug double conjugate, cytotoxic photo‐bystander effect, heterogeneous tumor‐antigen expression, near‐infrared photoimmunotherapy, photo‐inducible drug release

## Abstract

Ideal cancer treatments specifically target and eradicate tumor cells without affecting healthy cells. Therefore, antibody‐based therapies that specifically target cancer antigens can be considered ideal cancer therapies. Antibodies linked with small‐molecule drugs (i.e., antibody–drug conjugates [ADCs]) are widely used in clinics as antibody‐based therapeutics. However, because tumors express antigens heterogeneously, greater target specificity and stable binding of noncleavable linkers in ADCs limit their antitumor effects. To overcome this problem, strategies, including decreasing the binding strength, conjugating more drugs, and targeting tumor stroma, have been applied, albeit with limited success. Thus, further technological advancements are required to remotely control the ADCs. Here, we described a drug that is photo‐releasable from an ADC created via simple double conjugation and its antitumor effects both on target and nontarget tumor cells. Specifically, noncleavable T‐DM1 was conjugated with IR700DX to produce T‐DM1‐IR700. Although T‐DM1‐IR700 itself is noncleavable, with NIR‐light irradiation, it can release DM1‐derivatives which elicited antitumor effect in vitro mixed culture and in vivo mixed tumor model which are mimicking heterogeneous tumor‐antigen expression same as real clinical tumors. This cytotoxic photo‐bystander effect occurred in various types mixed cultures in vitro, and changing antibodies also exerted photo‐bystander effects, suggesting that this technology can be used for targeting various specific cancer antigens. These findings can potentially aid the development of strategies to address challenges associated with tumor expression of heterogeneous antigen.

## 1 INTRODUCTION

Controlled and localized targeted drug delivery is ideal for minimizing systemic toxicity and inducing highly localized therapeutic effects.^1^ Because antibody–drug conjugates (ADCs) can selectively deliver drugs to cells expressing a target antigen, they are considered an ideal modality for treating various diseases, especially cancer.^2^, ^3^, ^4^ However, ADCs can get degraded before reaching the target site due to linker instability, resulting in reduced efficacy against the targets and increased systemic toxicity.^4^, ^5^, ^6^ Moreover, the pharmacokinetics and biodistribution of ADCs can be affected if multiple drugs are conjugated to the antibody, resulting in ADC degradation in the liver.^7^ Therefore, it is important to use appropriate linkers and drug‐to‐antibody ratios (DARs). The linkers between antibodies and drugs in clinically administered ADCs remain stable in the plasma when these ADCs have DARs of three or four.^2^, ^8^ Furthermore, stable linkers enhance the specificity of antibodies, thereby reducing the systemic toxicity of ADCs, which is called as a noncleavable linker. Although ADCs have shown success in treating blood cancers, their success has been limited against solid tumors. Unlike hematological malignancies, solid tumors are difficult to target, because the target antigens are heterogeneously expressed by tumor cells.^9^, ^10^ Thus, further technological advancements are required to develop remotely controlled ADCs that accumulate in targeted tumor lesions and widely release the drugs therein to eradicate both target and nontarget tumor cells.^11^, ^12^, ^13^

Trastuzumab (Tra) emtansine (T‐DM1) is a clinically applied ADC containing Trastuzumab (Tra), an HER2‐targeting humanized monoclonal antibody (mAb).^14^ Tra is covalently linked to the cytotoxic agent DM1 (a maytansinoid, cytotoxic component that binds to the ends of microtubules) with an *N*‐succinimidyl 4‐(*N*‐maleimidomethyl)cyclohexane‐1‐carboxylate (SMCC) linker (a thioether‐linked, noncleavable linker). T‐DM1 is catabolized in lysosomes after receptor‐mediated internalization by HER2‐expressing cancer cells, resulting in the release of DM1‐containing catabolites that subsequently bind to tubulin and cause mitotic arrest and apoptosis.^4^ Due to the noncleavable linker (SMCC) with thiol band in T‐DM1, this ADC is cytotoxic only after cellular internalization, and thus, does not exert bystander cytotoxic effects on nontarget cells.^3^, ^15^, ^16^ Therefore, T‐DM1 cannot fully eradicate solid tumors due to heterogeneous HER2 expression.^3^, ^16^

Near‐infrared (NIR)‐photoimmunotherapy (PIT) is a recently developed cancer therapy that involves exposing an antibody–photoabsorber conjugate to NIR light.^17^ An antibody–photoabsorber conjugate comprises a cancer cell‐specific mAb that is covalently conjugated to IRDye 700DX NHS ester (IR700), a silica‐phthalocyanine‐derived photoabsorber that binds the cell‐surface target antigen and induces necrosis after exposure to 690 nm NIR light.^18^, ^19^, ^20^, ^21^ This novel therapy is currently undergoing an international phase III clinical trial and was recently conditionally approved by the Pharmaceuticals and Medical Devices Agency (PMDA) in Japan.

Recent research revealed that the mechanism of NIR‐PIT was rapid necrotic cell death due to photochemical ligand reactions of IR700.^22^, ^23^, ^24^ This photochemical reaction changes the hydrophilic side chains (silanol) of IR700 into hydrophobic, which introduces the aggregation of the antibody‐IR700 conjugates. With this unique mechanism for specific cell membrane ruptures, NIR‐PIT is thought to be a new modality in cancer therapy. However, since NIR‐PIT exploited the antibody targeting ability, its highly selective cytotoxicity on the target‐expressing cells limits on the whole antitumor effect on heterogeneous antigen‐expressing tumors.^25^, ^26^

**FIGURE 1:**
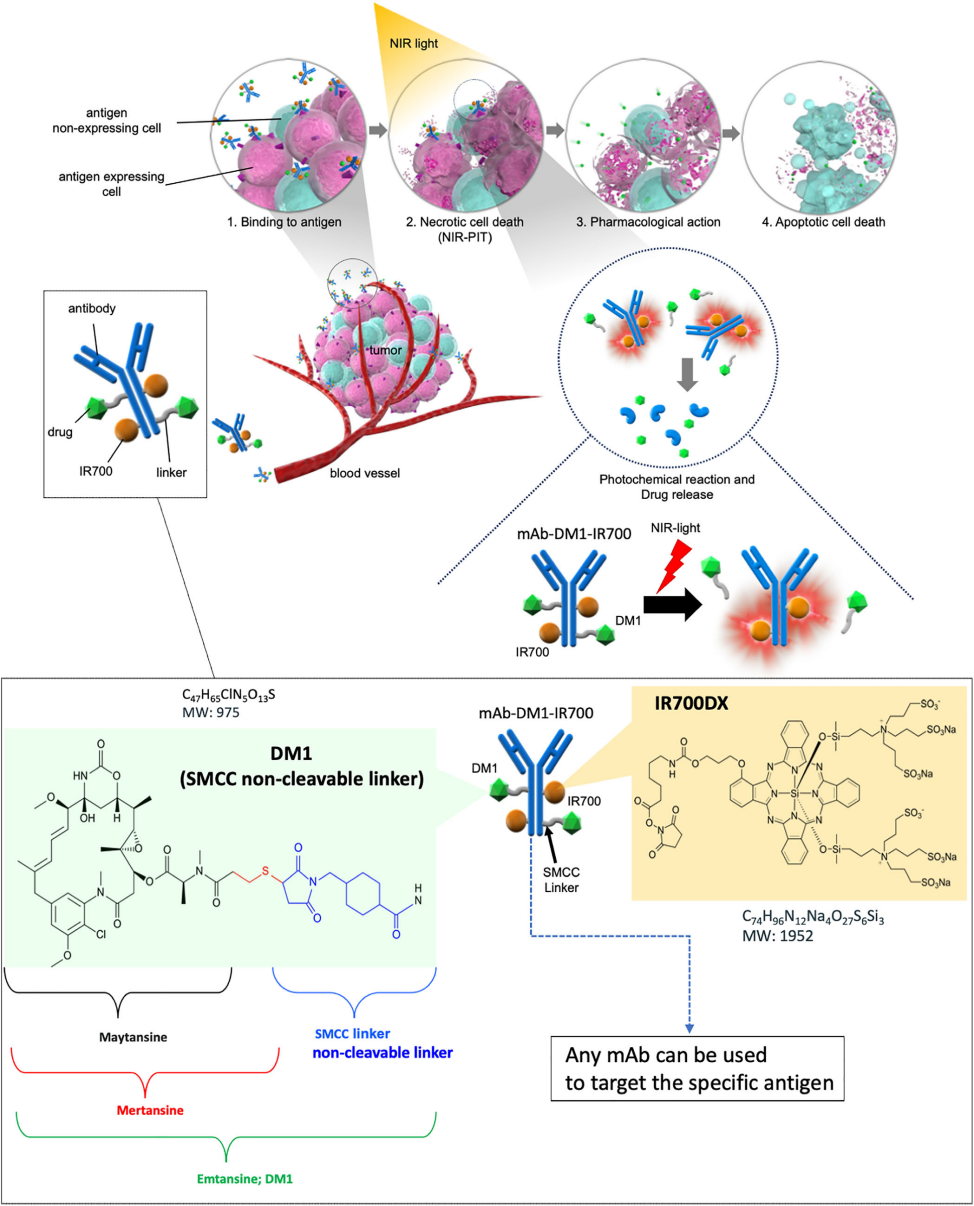
Scheme of NIR‐triggered drug release exerting a cytotoxic photo‐bystander effect on a mixed tumor (mimicking a tumor that expresses heterogeneous target antigens) and Schematic representation of mAb‐DM1–IR700 (double‐conjugated antibody). Schematic of the cytotoxic photo‐bystander effect of photo‐triggered drug release and an ADC conjugated to IR700. First, the dual conjugate (the ADC conjugated to IR700) was prepared for intravenous injection. The agents then accumulated near the targeted tumor antigen inside the tumor, which heterogeneously expressed the targeted antigens. Upon NIR‐light exposure, the targeted tumor cells were ruptured via NIR‐PIT (photo‐necrosis). At the same time, the conjugated drugs were released around the ruptured cells, and the drugs released in response to the photochemical reaction scattered to nontargeted tumor cells and induced cytotoxicity in the remaining live tumor cells. Schematic representation of mAb‐DM1**–**IR700. DM1 was linked to any mAb with the noncleavable thiol based SMCC linker. mAb‐DM1 was then double‐conjugated with IR700 to generate mAb‐DM1**–**IR700.

In this study, we created a simple double‐conjugated ADC comprising T‐DM1 and IR700 to enable remotely controlled drug release from ADCs (Figure 1). The T‐DM1‐IR700 released DM1‐derivatives via NIR‐light irradiation. The changing the antibody could also work this NIR‐light triggered drug release. The released DM1‐derivatives induced cytotoxicity on nontargeting tumor cells in vitro mixed cell culture and in vivo mixed tumor, mimicking heterogeneous antigen expressing tumors. After accumulation in target cells, the double‐conjugated ADC released the cytotoxic drug by NIR‐light and simultaneously induced necrosis in response to NIR‐light (NIR‐PIT effect), resulting in the eradication of both target and nontarget tumor cells. These results effectively demonstrated the concept of a “cytotoxic photo‐bystander effect” in mixed tumors (Figure 1).

## 2 RESULTS

### 2.1 Production of T‐DM1–IR700 and Tra–IR700

**FIGURE 2:**
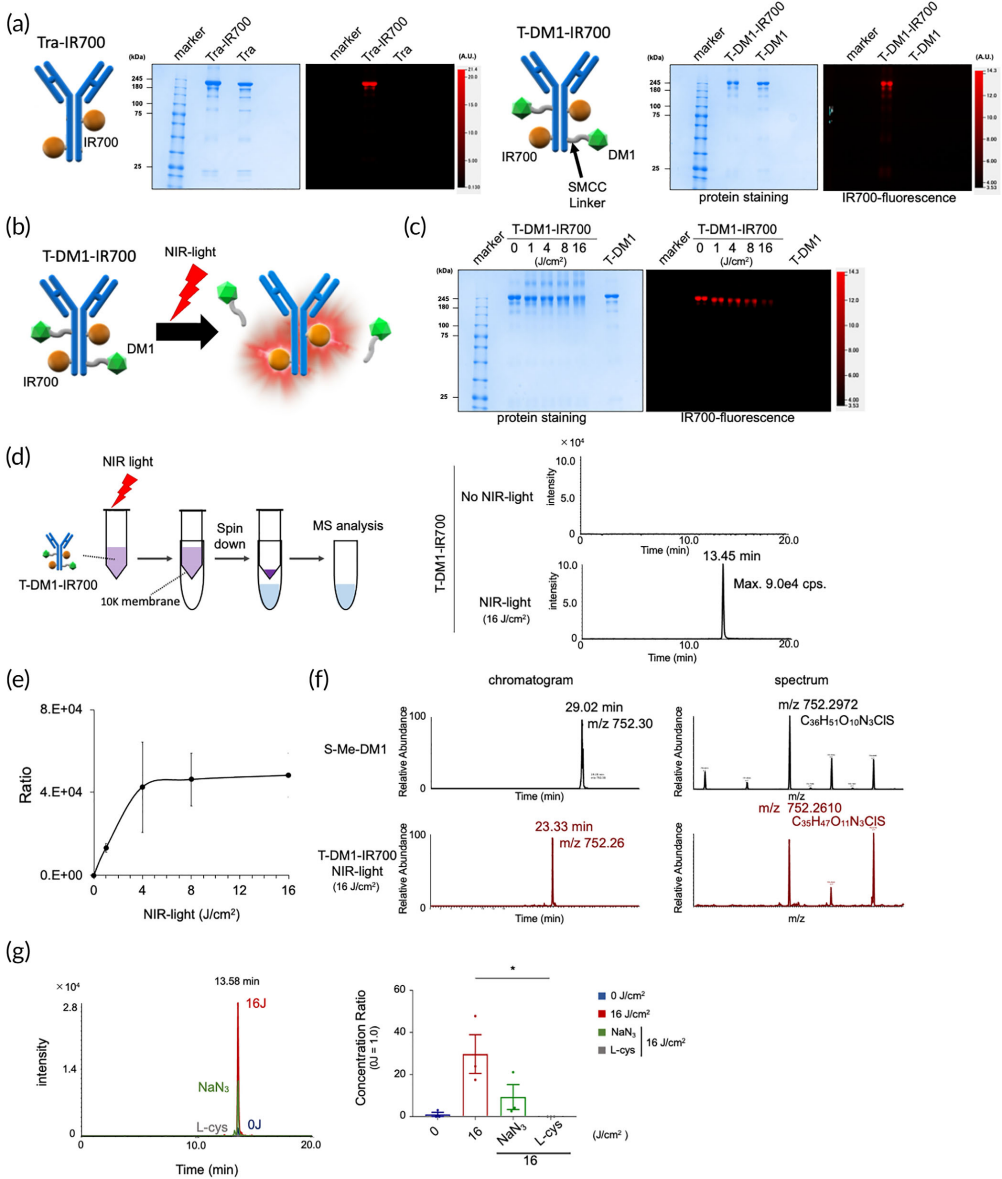
Photo‐triggered drug release from T‐DM1–IR700. (a) Validation of Tra–IR700 and T‐DM1–IR700 using SDS‐PAGE (left: Colloidal Blue protein staining; right: FLI at 700 nm). Diluted Tra or T‐DM1 was used as control. IR700 fluorescence was detected in the bands for Tra–IR700 and T‐DM1–IR700, suggesting that the conjugations were successful. (b) Schematic of our hypothesis of how NIR light triggers DM1 release from T‐DM1–IR700. (c) SDS‐PAGE of NIR‐light‐irradiated T‐DM1–IR700. (d) Schematic of MS sample preparation (left) and LC–MS/MS data showing a specific peak in a tube irradiated with NIR light (16 J/cm^2^; right). (e) Relative volume of the detected peak, as measured using MS (*n* = 3). (f) Product ion analysis of the irradiated sample (16 J/cm^2^) using high‐resolution mass spectrometer. The mass spectral fragmentation pattern of T‐DM1‐IR700 was matched to that of S‐Me‐DM1. Further fragmentation analysis was shown in Figure S1. (g) MS data obtained following inhibition of the specific peak (DM1) in an NIR‐light‐irradiated (16 J/cm^2^) tube. The relative ratio (defined 0 J/cm^2^ tube = 1) is shown (*n* = 3). The data represent the mean ± standard deviation. *p < 0.05 (Kruskal–Wallis test with Dunn's post hoc test)

In T‐DM1, Tra is conjugated to the maytansinoid DM1—a cytotoxic tubulin inhibitor—,^27^ which comprises an SMCC linker (noncleavable) and mertansine (a thiol‐containing maytansinoid) (Figure 1). These structures facilitate the endocytosis of T‐MD1 after HER2 binding, followed by its degradation and the intracellular release of DM1.^4^, ^14^ In this study, we conjugated T‐DM1 to IR700 to generate the simple double‐conjugate T‐DM1–IR700 (Figure 2a), whose release was then evaluated. Trastuzumab‐IR700 (Tra–IR700) was also produced as a control with the conjugation of trastuzumab and IR700. The successful conjugation of Tra and T‐DM1 to IR700 was confirmed using sodium dodecyl sulfate polyacrylamide gel electrophoresis (SDS‐PAGE) and IR700‐based fluorescence imaging (FLI) (Figure 2a). The number of IR700‐dye to mAb molecules was adjusted so that approximately three IR700 molecules were conjugated on per one mAb molecule.

### 2.2 NIR‐light triggered drug releasing from T‐DM1–IR700

We first characterized the NIR‐light triggered drug releasing from T‐DM1‐IR700 (Figure 2b). Cytotoxicity on NIR‐PIT is based on a photochemical reaction involving silanol ligand release, which makes hydrophilic conjugates hydrophobic and results in their aggregation.^22^, ^28^ Previous data demonstrated that silicon phthalocyanines undergo axial ligand exchange especially in hypoxic conditions and generate toxic reactive oxygen species (ROS) especially in the normoxic conditions.^29^, ^30^ SDS‐PAGE revealed the formation of nonfluorescent, irradiated T‐DM1–IR700 aggregates with sizes larger than those of IgG; however, IR700‐fluorescence was dose‐dependently quenched by NIR‐light (Figure 2c). Thus, after NIR irradiation, T‐DM1–IR700 became aggregated and lost IR700‐fluorescence.

We then analyzed the photo‐release of DM1 derivatives from the T‐DM1–IR700 conjugates in tube (Figure 2d, left panel). Since the T‐DM1 is based on noncleavable thiol linker, no specific peak was detected from the supernatants without NIR‐light (Figure 2d). After NIR‐light irradiation (16 J/cm^2^), a specific peak was detected by high‐performance liquid chromatography (Figure 2d), and the relative volume of photo‐released substrates was dose‐dependently elevated by NIR‐light, and a plateau was observed at 8 J/cm^2^ (Figure 2e).

We then compared the substrates from the irradiated sample with S‐ME‐DM1 using mass spectroscopy (MS) and identified the substrates as DM1 derivatives by product ion analysis using a high‐resolution mass spectrometer with further fragmentation analysis (Figures 2f and S1).^31^ Additionally, we performed LC–MS/MS with aMFc‐DM1‐IR700 (with a different antibody) and successfully detected photo‐released substrates. Each peak has similar retention time, suggesting that they were DM1 derivatives (Figure S2). Collectively, these results identified the DM1 derivatives were photo‐released from T‐DM1–IR700. Specifically, the photo‐release reaction was universal, irrespective of the changes in the antibody.

To determine whether the releasing the DM1 derivatives depended on NIR‐light triggered silanol release from IR700,^29^, ^30^ or ROS generation from IR700 with NIR‐light, we added an ROS quencher (sodium azide [NaN_3_]) or an electron donor (l‐cysteine) to the tube and evaluate the relative volume of photo‐released substrates (Figure 2g). We found that the relative volume of the photo‐released DM1 derivatives was partially decreased in the presence of NaN_3_ and almost fully blocked with l‐cysteine, suggesting that ROS generation was primarily involved in DM1 derivatives' photo‐release with cleavage of the linker.

### 2.3 In vitro assessment of NIR‐PIT with Tra–IR700 and T‐DM1–IR700, and “cytotoxic photo‐bystander effect” from the released DM1 derivatives on in vitro mixed cell culture

**FIGURE 3:**
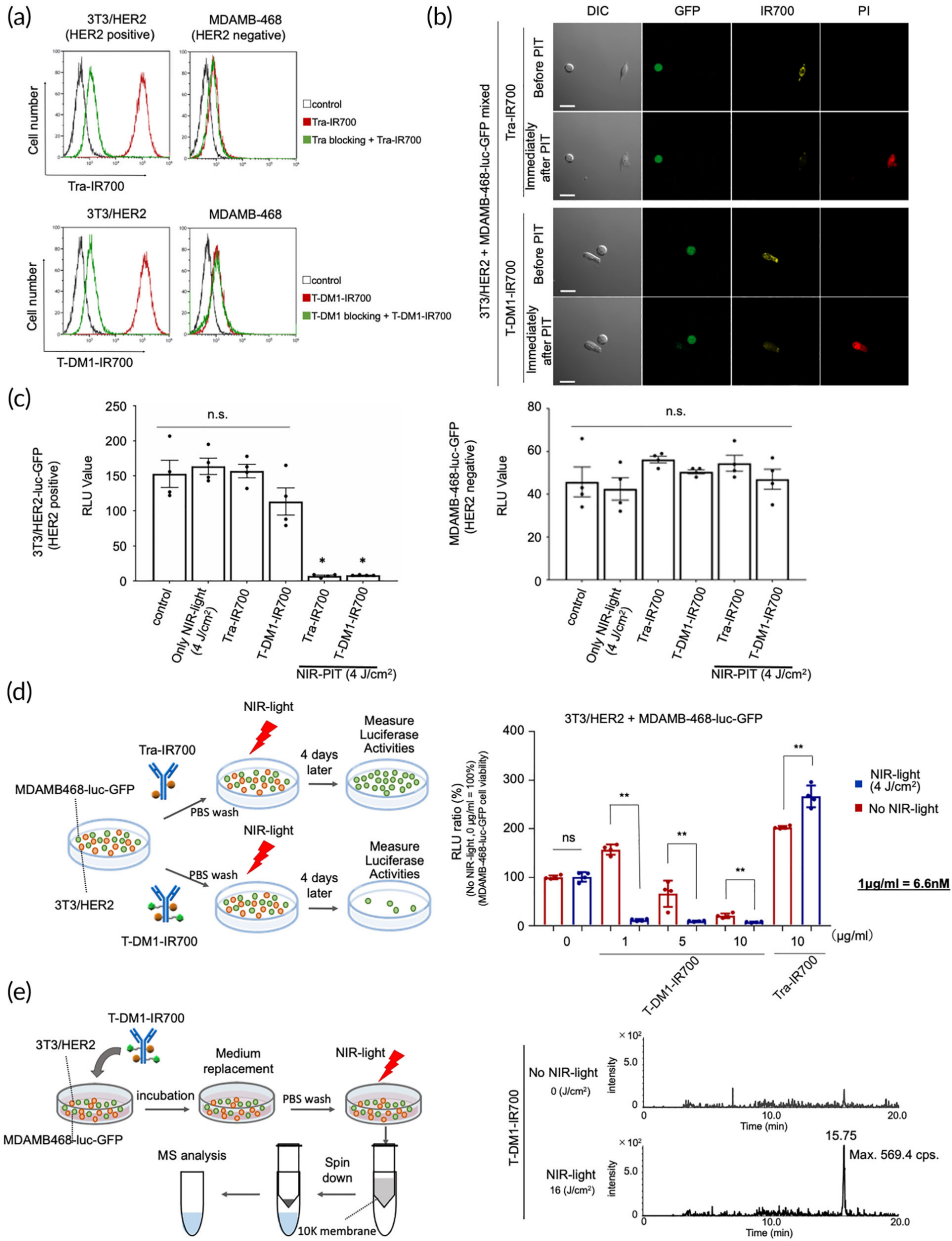
in vitro evaluation of the NIR‐PIT and cytotoxic photo‐bystander effects via photo‐triggered drug release from T‐DM1–IR700. (a) Flow cytometric analysis of the binding of Tra–IR700 and T‐DM1–IR700 to HER2+ (3T3/HER2) and HER2−(MDAMB‐468) cells. Preincubation with excess Tra or T‐DM1 inhibited the binding of Tra–IR700 or T‐DM1–IR700 to 3T3/HER2 cells, respectively, indicating that Tra–IR700 and T‐DM1–IR700 specifically bound to the HER2 antigen. Neither Tra–IR700 nor T‐DM1–IR700 showed IR700 fluorescence signals in the presence of HER2−MDAMB‐468 cells. (b) Microscopic observations before and immediately after HER2‐targeted NIR‐PIT. Mixed cultures of HER2+ 3T3/HER2 and HER2− MDAMB‐468‐luc‐GFP cells were incubated with Tra–IR700 or T‐DM1–IR700 overnight and observed under a microscope before and immediately after irradiation with NIR light (4 J/cm^2^). Necrotic cell death (revealed using PI staining) was observed only for HER2+ 3T3/HER2 cells after NIR light exposure, whereas HER2− MDAMB‐468‐luc‐GFP cells remained intact. Confirmation of the selective cytotoxicity induced by NIR‐PIT with both Tra–IR700 and T‐DM1–IR700. Scale bars: 20 μm. (c) In vitro NIR‐PIT (4 J/cm^2^) with Tra–IR700 (1 μg/ml) or T‐DM1–IR700 (1 μg/ml) on HER2+ (3T3/HER2‐luc‐GFP) and HER2− (MDAMB‐468‐luc‐GFP) cells. Luciferase activities were measured as RLU values (*n* = 4, *p < 0.001). (d) Co‐culture of 3T3/HER2 and MDAMB‐468‐luc‐GFP cells. NIR‐PIT was performed following treatment with T‐DM1–IR700 (1, 5, or 10 μg/ml) or Tra–IR700 (10 μg/ml), after which the mixed cultures were incubated for 4 days (left panel). Luciferase activities were measured as RLUs, and the viability of nontargeted MDAMB‐468‐luc‐GFP cells was measured 4 days after NIR‐light irradiation. Upon NIR‐PIT following Tra–IR700 treatment of the mixed culture, HER2+ 3T3/HER2 cells were eradicated, resulting in more space available for the nontargeted MDAMB‐468‐luc‐GFP cells to grow (*n* = 4). NIR‐PIT showed no immediate effect on MDAMB‐468‐luc‐GFP cells in the mixed culture (Figure S5). Only MDAMB‐468‐luc‐GFP cells treated with T‐DM1–IR700 did not show any significant decrease in luciferase activity 4 days after irradiation (Figure S6). Other cell‐line combinations (HER2+ and HER2− or ‐low cells) were also examined and showed cytotoxic photo‐bystander effects (Figure S8). In panels (e) and (f), the data are presented as the mean ± standard error of the mean (SEM). In panel (g), the data are presented as the mean ± standard deviation. *p < 0.0001, **p < 0.01 (Student's *t* test). (e) Schematic depicting the MS‐analysis procedure for investigating the supernatants of mixed 3T3/HER2 and MDAMB‐468‐luc‐GFP cells treated with T‐DM1–IR700‐mediated NIR‐PIT (left) and LC–MS/MS data showing a specific peak only in an NIR‐light‐irradiated (16 J/cm^2^) tube (right).

Next, Tra–IR700 and T‐DM1–IR700 bound to HER2‐expressing 3T3/HER2 cells (HER2+), whereas these binding events were blocked by excess Tra or T‐DM1, respectively, indicating that they specifically bound to HER2 (Figure 3a). Neither Tra‐IR700 nor T‐DM1‐IR700 bound to HER2− MDAMB‐468 cells (Figure 3a).

We then confirmed the effects of NIR‐PIT on 3T3/HER2 cells bound by Tra–IR700 or T‐DM1–IR700 via microscopic observations, before and immediately after NIR‐light irradiation (Figure 3b). Following exposure to NIR light (4 J/cm^2^), we observed HER2+ 3T3/HER2 cell necrosis via propidium iodide (PI) staining, whereas HER2− MDAMB‐468‐luc‐GFP cells remained intact (Figure 3b). Co‐culture of HER2− and HER2+ cells revealed that NIR‐PIT with Tra–IR700 or T‐DM1–IR700 specifically induced necrosis in the mixed in vitro culture.

Along with the expression, in vitro NIR‐PIT (4 J/cm^2^) with Tra‐IR700 or T‐DM1‐IR700 induced significant cytotoxicity on 3T3/HER2‐luc‐GFP (HER2+), whereas that did no significant cytotoxicity on MDAMB‐468‐luc‐GFP (HER2‐) (*n* = 4, *p < 0.001, compared to control) (Figure 3c). NIR‐light (4 J/cm^2^), Tra–‐IR700 or T‐DM1–IR700 alone demonstrated no significant difference to control in vitro 3T3/HER2‐luc‐GFP (HER2+) or MDAMB‐468‐luc‐GFP (HER2‐), respectively.

To evaluate the toxicity of T‐DM1–IR700 toward HER2+ cells, we measured cell viabilities 4 days after exposure to S‐methyl‐DM1 (S‐Me‐DM1) or T‐DM1–IR700. Exposure to S‐Me‐DM1 resulted in the death of 3T3/HER2‐luc‐GFP and MDAMB‐468‐luc‐GFP cells, with half‐maximal inhibitory concentration (IC_50_) values of 3 and 0.3 nM, respectively (Figure S3). On exposing other cell lines (H2170, Calu‐3, H1975, and MDAMB‐231) to S‐Me‐DM1, we observed cytotoxicity in all cases (IC_50_ values: ~0.1–10 nM in various cell lines; Figure S3). In contrast, T‐DM1–IR700 induced cell death in 3T3/HER2‐luc‐GFP cells, with an IC_50_ of ~2.5 nM, whereas ~100 nM T‐DM1–IR700 induced cell death in MDAMB‐468‐luc‐GFP cells (Figure S4). Moreover, Tra–IR700 inhibited the growth of 3T3/HER2‐luc‐GFP cells at a high concentration (>10 nM) without impacting the growth of MDAMB‐468‐luc‐GFP cells. Collectively, these data confirmed the specific toxicity of T‐DM1–IR700 toward HER2‐expressing cells and S‐Me‐DM1 with the suitable concentration could induce cytotoxicity to several cancer cell lines.

We then evaluated the effects of NIR‐PIT (4 J/cm^2^) with Tra–IR700 or T‐DM1–IR700 on an in vitro mixed culture of HER2+ 3T3/HER2 and HER2− MDAMB‐468‐luc‐GFP cells (Figure 3d). Immediately after NIR‐light irradiation, neither Tra–IR700‐ nor T‐DM1–IR700‐mediated NIR‐PIT exerted an effect on nontargeted MDAMB‐468‐luc‐GFP cells in the mixed culture (Figure S5). This specific photocytotoxicity was consistent with that described previously.^18^, ^32^

MDAMB‐468‐luc‐GFP cells treated with T‐DM1–IR700 did not show any significant decrease in luciferase activity at 4 days after irradiation (Figure S6). No in vitro significant effects on nontargeting cells (MDAMB‐469‐luc‐GFP) in mixed culture was detected with T‐DM1 incubation, and a mixture of Tra‐IR700 and T‐DM1 with NIR‐light grew nontargeting cells more than that without NIR‐light or control. NIR‐PIT with Tra‐IR700 and T‐DM1 (NIR‐PIT) eradicated 3 T3/HER2 cells from the mixed culture, resulting in diminishing contact inhibition (making more space), which grew nontargeting cells (MDAMB‐468‐luc‐GFP) more (Figure S7). Four days after NIR‐PIT, Tra–IR700‐mediated NIR‐PIT eradicated most of the 3T3/HER2 cells, resulting in making more new space, thereby allowing the MDAMB‐468‐luc‐GFP cells to grow more than that observed under control conditions. Moreover, Tra–IR700 alone inhibited the growth of 3T3/HER2 cells to facilitate more space to grow MDAMB‐468‐luc‐GFP cells than that observed with the controls. Furthermore, the relative light unit (RLU) ratio of Tra–IR700‐mediated NIR‐PIT was greater than that of Tra–IR700 alone, suggesting that NIR‐PIT almost killed 3T3/HER2 cells (*n* = 4, *p* = 0.0013). These data suggested that Tra–IR700‐mediated NIR‐PIT eradicated only 3T3/HER2 cells without affecting HER2− MDAMB‐468‐luc‐GFP cells (Figure 3d).

Intriguingly, in a mixed culture, T‐DM1–IR700‐mediated NIR‐PIT exerted toxicity toward HER2− MDAMB‐468‐luc‐GFP cells at 4 days after NIR‐light irradiation (Figure 3d). Notably, we observed that the cytotoxicity of NIR‐PIT was significantly higher at concentrations ranging from 1 μg/ml (6.6 nM) to 10 μg/ml (66 nM) (*p* < 0.0001 at 1 mg/ml; *p* < 0.001 at 5 and 10 μg/ml) (Figure 3d) relative to that obtained without NIR‐light exposure. We designated this effect as “cytotoxic photo‐bystander effect,” which describes toxicity toward nontargeted cancer cells after NIR‐light irradiation due to the photo‐released DM1 derivatives. The photo‐released DM1 derivatives were also detected in the supernatants of T‐DM1–IR700‐mediated NIR‐PIT‐treated 3T3/HER2 and MDAMB‐468‐luc‐GFP mixed‐culture cells (Figure 3e). Furthermore, in various mixed (HER2‐overexpressing, HER2‐low, or HER2−) cell cultures, the cytotoxic photo‐bystander effect was observed on nontargeted HER2− or HER2‐low cells (Figure S8), suggesting that this effect of T‐DM1–IR700‐mediated NIR‐PIT exerted across the cancer cell types.

### 2.4 HER2 expressed heterogeneously in human lung cancer specimens

To test HER2 expression in lung cancer, 14 nonsmall cell lung adenocarcinoma (NSCLAC), 12 NSCL squamous cell cancer (NSCLSC), and five SCL cancer (SCLC) samples were obtained and immunostained with HER2 antibodies. Seven of the 14 NSCLACs, one of the 12 NSCLSCs, and none of the SCLC samples were HER2‐positive (Figure S9A,B). Additionally, all HER2+ NSCLAC samples showed heterogeneous expression of HER2 inside the tumors. These data support the motivation for developing a technology for overcoming tumor heterogeneity with the NIR‐light triggered drug releasing. Therefore, we made mixed tumor model mimicking this heterogeneous expression of HER2 in human lung cancer resected specimens.

### 2.5 Cytotoxic photo‐bystander effect on in vivo

**FIGURE 4:**
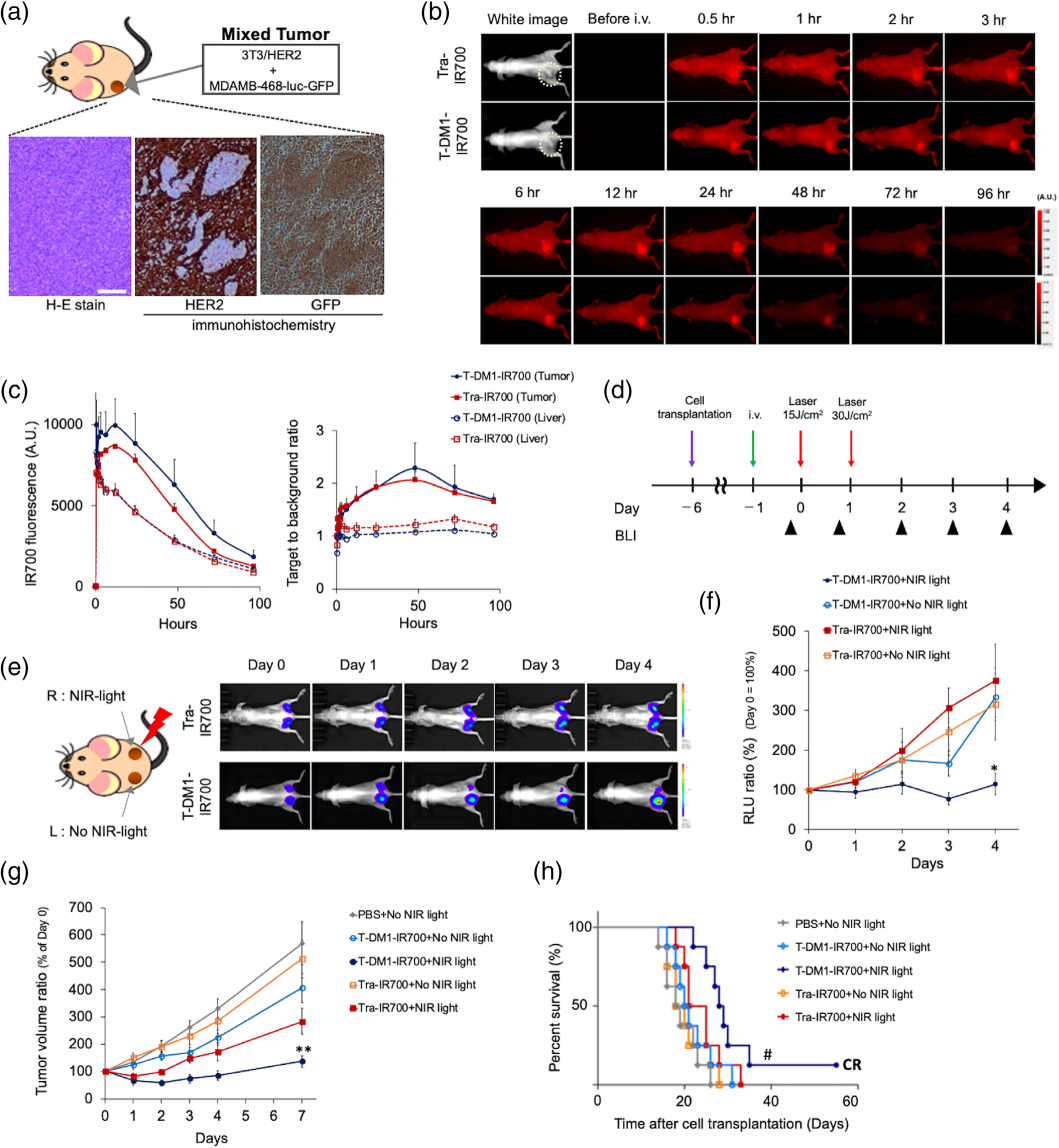
Evaluation of the in vivo cytotoxic photo‐bystander effect. (a) Tumors excised 6 days after the inoculation of nude mice with HER2+ 3T3/HER2 cells and HER2− MDAMB‐468‐luc‐GFP cells. Scale bar: 300 μm. The tumor sample was imuunostained with HER2 or GFP antibody. (b) Representative IR700 fluorescence images of Tra–IR700 and T‐DM1–IR700‐injected mice. We used the mice tumor model with over 1 cm tumors. (c) Fluorescence intensity measurements of the tumor and liver. The target‐to‐background ratios of the tumor and liver are indicated (*n* = 3 mice). (d) In vivo therapeutic regimen involving tumor cell inoculation, Tra–IR700 or T‐DM1–IR700 injection, and NIR‐light exposure. BLI was performed at the indicated points (arrowheads). BLI indicated HER2− nontargeted MDAMB‐468‐luc‐GFP tumor activity in the mixed tumor. (e) Mixed tumors inoculated on both dorsa of mice, with only the right‐sided tumor irradiated with NIR light. Representative BLI of right‐sided NIR‐PIT with Tra–IR700 or T‐DM1–IR700 is shown. (f) Quantitative RLUs, indicating nontargeted HER2− MDAMB‐468‐luc‐GFP cells inside the mixed tumors (*n* = 6 mice/group). (g) Mixed tumor volume (mm^3^) of the ratio (defined as day 0 = 100; *n* = 8 mice/group). (h) Survival of HER2‐targeted NIR‐PIT with T‐DM1–IR700 or Tra‐IT700 on mixed tumors (*n* = 8 mice/group). In panel (c), the data represent the mean ± SEM. In panel (f), *p = 0.033 < 0.05 (Kruskal–Wallis test with Dunn's post hoc test). In panel (g), **p = 0.0005 < 0.001 (Kruskal–Wallis test with Dunn's post hoc test). In panel (h), #*p* = 0.035 < 0.05 (log‐rank test)

To confirm the in vivo cytotoxic photo‐bystander effect of NIR‐PIT with T‐DM1–IR700, we established a mixed tumor model comprising HER2+ 3T3/HER2 and HER2− MDAMB‐468‐luc‐GFP cells, and the antitumor cytotoxic photo‐bystander effect (nontargeted tumor cells) in this mixed tumor model was monitored by measuring the luciferase activity of MDAMB‐468‐luc‐GFP cells. First, we evaluated the mixed tumor using histology and immunostaining (Figure 4a). HER2 or GFP immunostaining revealed that the mixed tumors were well mixed, with random HER2+ and HER2− (GFP+) lesions, indicating successful establishment of the mixed tumor model. We then examined the biodistribution of Tra–IR700 and T‐DM1–IR700 in the mixed tumors using IR700‐FLI (Figure 4b), with the Tra–IR700 and T‐DM1–IR700 doses (3.6 mg/kg) according to the dose, which is used clinically for humans.

After administering these doses via injection, we observed high IR700 fluorescence throughout the bodies of mice, with tumors gradually becoming visible during the following 3 h (Figure 4b). The highest fluorescence intensity in the tumors was observed 12 h after injection, whereas the highest tumor‐to‐background ratio was observed on Days 1 through 3 (Figure 4c). We did not detect the specific localization of IR700 fluorescence in tissues other than liver and urinary bladder, presumably owing to hepatic metabolism and urinary excretion. The Tra–IR700: T‐DM1–IR700 fluorescence ratios did not differ significantly at any time point, suggesting that their biodistributions were almost entirely dependent on Tra and that the effects of the conjugated drugs were negligible (Figure 4c). Ex vivo biodistribution study revealed that lung and heart had some IR700‐fluorescence until 12 h compared to tumors or livers. The Tra–IR700 or T‐DM1–IR700 fluorescence on ex vivo tumors did not differ significantly at any time point (Figure S10). The analysis of frozen tumor section revealed that Tra–IR700 or T‐DM1–IR700 specifically accumulated on the HER2 expressing tumor regions (non‐GFP regions) in mixed ex vivo tumors (Figure S11). These data indicated that Tra–IR700 and T‐DM1–IR700 were specifically accumulated in mixed tumors, and that NIR‐light irradiation 1–2 days postadministration might be appropriate. Moreover, IR700 fluorescence could be used as a marker to indicate the site of NIR‐light irradiation.

We then tested the in vivo cytotoxic photo‐bystander effects on mixed tumors (Figure 4d). Only T‐DM1 administration had no in vivo cytotoxic bystander effect on the mixed tumors (Figure S12). To precisely compare the in vivo cytotoxic photo‐bystander effects induced by T‐DM1–IR700‐mediated NIR‐PIT, we developed a bilateral mixed‐tumor mouse model, and only the right side of the mixed tumor was irradiated with NIR light (Figure 4e, left). In the mouse model injected with Tra–IR700, there was a gradual increase in luciferase activity with no antitumor effect on nontarget tumor populations on either the right side with or without light irradiation. Furthermore, the nontargeted cell population on the left side of the mixed tumor in T‐DM1–IR700‐injected mice gradually grew, which was confirmed by the increased luciferase activity seen in bioluminescence imaging (BLI). However, the BLI intensity of the right side of the mixed tumor in T‐DM1–IR700‐injected mice decreased with NIR‐light irradiation, indicating in vivo cytotoxic photo‐bystander effects (Figure 4e). Quantitative analyses of luciferase activity (the non‐NIR‐PIT‐targeted MDAMB‐468‐luc‐GFP population in the mixed tumor) revealed that only T‐DM1–IR700‐mediated NIR‐PIT exerted antitumor effects on the MDAMB‐468‐luc‐GFP population in the mixed tumors, whereas other treatments showed no remarkable effects (T‐DM1–IR700 + NIR light versus Tra–IR700 + NIR light on day 3; *p = 0.033, Kruskal–Wallis test with Dunn's post hoc test; Figure 4f). Moreover, quantification of tumor volumes to compare the antitumor effects on whole mixed tumors indicated that T‐DM1–IR700‐mediated NIR‐PIT exerted a strong antitumor effect on whole mixed tumors, suggesting its antitumor effects on both the target 3T3/HER2 cells and nontarget MDAMB‐468‐luc‐GFP cells and confirming the in vivo cytotoxic photo‐bystander effect (**p = 0.0005, Kruskal–Wallis test with Dunn's post hoc test, *n* = 8 mice per group) (Figure 4g). Furthermore, survival was significantly prolonged in the T‐DM1–IR700 NIR‐PIT group (#*p* = 0.035, log‐rank test; Figure 4h), with achievement of a complete response (CR). We also evaluated in vivo cytotoxic photo‐bystander effects on mixed tumors composed of Calu‐3 and MDAMB‐468‐luc‐GFP cells, and in vivo cytotoxic photo‐bystander effects were also exerted on nontargeted MDAMB‐468‐luc‐GFP tumors (Figure S13). Collectively, these data demonstrated the in vivo cytotoxic photo‐bystander effects of T‐DM1–IR700‐mediated NIR‐PIT via photo‐triggered releasing of DM1 derivatives, the antitumor effect of NIR‐PIT, and cytotoxic photo‐bystander effects on mixed targeted and nontargeted tumors.

## 3 DISCUSSION

In this study, we demonstrated a cytotoxic photo‐bystander effect induced in response to the photo‐triggered release of the drug from an IR700‐conjugated ADC, T‐DM1–IR700. This technology efficiently overcame the heterogeneity of tumor‐antigen expression, which is the one of the most difficult topics in cancer treatment. After intravenous injection of a double conjugate, the agents accumulated at the target tumor site and bound cells expressing the target antigen inside the heterogeneous tumor. Upon exposure to NIR light, the targeted tumor cells ruptured due to the NIR‐PIT effect along with simultaneous release of the drugs from ADCs. The photo‐released drugs then spread to nontargeted tumor cells and exerted a cytotoxic effect on all tumor cells (Figure 1). This photo‐bystander cytotoxic effect is unspecific; therefore, the photo‐released cytotoxic drugs could give damage on adjacent normal cells. Moreover, in the tumor microenvironment, anti‐tumor immune cells have an important role for the effective immunotherapy. Unspecific photo‐bystander effect might affect these immune cells, which might be a concern for this technology of photo‐triggered drug releasing. However, the NIR‐light irradiation is done after the ADCs are specifically accumulated on targeted tumor site, the damage on normal cells could be controlled to be minimized.

We successfully developed an in vivo photo‐released drug system involving the use of NIR light. Drug release was spatiotemporally regulated using NIR light, which can penetrate tissues to a deeper extent than light of other wavelengths, without damaging the normal tissues. Additionally, we used ADCs to target tumor antigens and achieve accumulation in tumor lesions, resulting in the release of drugs at high concentrations upon NIR‐light irradiation of the tumor area. These findings indicated that drugs with high toxicity can be efficiently utilized. Interestingly, this method employs extremely simple chemistry for conjugating IR700 to ADCs, and we further demonstrated the effectiveness of the method using different antibodies; therefore, we could exploit this drug releasing technology for a variety of cancers. Notably, targeting molecules (peptides and/or ligands) can also be used in this approach. Furthermore, this method is easily translatable to the clinic, given that T‐DM1 has already been clinically approved,^15^, ^33^ and NIR‐PIT was recently approved by the PMDA in Japan. Finally, this method can be applied to fields other than those related to cancer therapy.

Concerns remain regarding the use of this treatment. First, we only demonstrated the efficacy of SMCC‐DM1 combinations with antibodies conjugated to IR700; therefore, further studies are required using other combinations to optimize the linkers and drugs that can be utilized. Moreover, the penetration of NIR light in the body is somewhat limited. Although there are reports on light sources attached to endoscopes and implantable devices,^34^, ^35^ it is crucial to develop flexible medical light sources. Lastly, the distribution of the drug released by the NIR‐light is unknown, and the antitumor effect might be limited. However, since a sufficient amount of drug is accumulated in the tumor by the targeted nature of the ADC, the concentration of the released drug would be high enough to have a sufficient effect.

## 4 CONCLUSION

In conclusion, we demonstrated a system involving NIR‐light‐triggered drug release in a heterogeneous tumor for efficient eradication of tumor cells. The accumulation of IR700 fluorescence of the photo‐absorber in conjugates could also be used as a noninvasive guide for determining the therapeutic area and as a biomarker to confirm the efficacy of therapy. Furthermore, as this system enables target switching by changing the mAbs or drugs, it can be used as a potential novel platform for photo‐controlled drug release. This technology can also be readily translated to clinical settings based on the recent approval of NIR‐PIT for therapeutic applications in 2020 and the current use of T‐DM1 in clinical practice.

## 5 MATERIALS AND METHODS

### 5.1 Study design

All in vivo experiments were performed in compliance with the Guide for the Care and Use of Laboratory Animal Resources of Nagoya University Animal Care and Use Committee (protocols approval numbers 2017‐29438, 2018‐30096, 2019‐31234, 2020‐20104, 2021‐20242, and 2022‐220370). The usage of materials (resected specimens) from patients (approval number 2017‐0487) was approved by the ethical board of Nagoya University, Clinical Research Committee (which conformed to the principles set out in the WMA Declaration of Helsinki and the Department of Health and Human Services Belmont Report). Patients provided informed consent for the use of their resected tumor samples in this study and were informed that they could withdraw the consent at any time if desired.

### 5.2 Xenograft tumor model

Female homozygous athymic nude mice (8–10 weeks old) were obtained from Chubu Kagaku Shizai (Nagoya, Japan). The mice were anesthetized with isoflurane during the injection procedures. A mixture of 3T3/HER2 (5 × 10^6^) and MDAMB‐468‐luc‐GFP (1 × 10^7^) cells was injected subcutaneously into the dorsum of each mouse. A mixture of Calu‐3 (1 × 10^7^) and MDAMB‐468‐luc‐GFP (1 × 10^7^) cells was injected subcutaneously into the dorsum of each mouse for another example of in vivo photo‐bystander effect (Figure S13). The greatest longitudinal diameter (length) and greatest transverse diameter (width) were measured to calculate the estimated tumor volumes, as follows: tumor volume = length × width^2^ × 0.5. Tumors approximately 200 mm^3^ at Day −1 (Figure 4d) were selected for the study. Mice were sacrificed using carbon dioxide inhalation when their tumor diameters reached 20 mm.

### 5.3 Cell lines and cell culture

HER2‐expressing mouse fibroblasts (3T3/HER2 cells) were established by transfecting an *HER2*‐expression plasmid into NIH/3T3 cells.^32^ HER2+ human breast cancer cells (SK‐BR‐3), HER2− human breast cancer cells (MDAMB‐468 and MDAMB‐231), lung cancer cells (Calu‐3 and H2170), HER2‐weakly positive lung cancer cells (H1975), and gastric cancer cells (N87) were obtained from American Type Culture Collection (Manassas, VA, USA).

Luciferase‐expressing cells were established via the transduction of RediFect Red‐FLuc‐Puromycin or RediFect Red‐FLuc‐GFP lentiviral particles^18^, ^36^ (PerkinElmer, Waltham, MA, USA). Stable and high luciferase expression was determined after >10 passages. All cell lines were cultured in RPMI‐1640 medium (Thermo Fisher Scientific, Waltham, MA, USA) supplemented with 10% heat inactivated fetal bovine serum, 100 IU/ml penicillin, and 100 μg/ml streptomycin (Thermo Fisher Scientific) at 37°C under an atmosphere of 5% CO_2_.

### 5.4 Reagents

A water‐soluble silicon phthalocyanine derivative and IR700 were purchased from LI‐COR Bioscience (Lincoln, NE, USA). Humanized Tra (Herceptin) and T‐DM1 (Kadcyla) IgG_1_ mAbs against HER2 were purchased from Chugai Pharmaceutical Co. (Tokyo, Japan). The anti‐human IgG Fc‐DM1 antibody with a noncleavable linker (αHFc‐NC‐DM1) was purchased from Moradec, LLC. (San Diego, CA, USA). S‐Me‐DM1 was purchased from Abcam (Cambridge, UK).

### 5.5 Synthesis of IR700‐conjugated Tra or T‐DM1

Tra (1.0 mg, 6.8 nmol), T‐DM1 (1.0 mg, 6.6 nmol), and αHFc‐NC‐DM1 (300 μg, 2.0 nmol) were mixed with IR700 NHS ester (66.8 μg, 34.2 nmol, 5 mM in DMSO) in Na_2_HPO_4_ buffer (pH 8.5; 0.1 M) at room temperature for 1 h. The mixture was purified using a Sephadex G50 column (PD‐10; GE Healthcare, Piscataway, NJ, USA).^37^ The protein concentration was confirmed using a Coomassie Plus Protein Assay Kit (Thermo Fisher Scientific) by measuring the absorption at 595 nm with a Novaspec Plus spectrophotometer (GE Healthcare).^38^ The concentration of IR700 was calculated based on the absorption at 689 nm to confirm the number of IR700 molecules conjugated to the antibody.^36^, ^39^ The number of mAb molecules was adjusted to approximately three IR700 molecules per one mAb molecule.

### 5.6 Sodium dodecyl sulfate polyacrylamide gel electrophoresis

SDS‐PAGE was performed using 4%–20% Tris‐Glycine mini gels (Thermo Fisher Scientific) to confirm mAb–IR700 conjugation.^40^, ^41^ The fluorescent bands were visualized with a Pearl Imager (LI‐COR Bioscience), using the 700 nm fluorescence channel after electrophoresis for 90 min at 20 mA. The gel was stained with Colloidal Blue (Thermo Fisher Scientific) to confirm the molecular weight of the mAb–IR700 conjugates.

### 5.7 Immunostaining

HER2 immunostaining was performed on surgically resected specimens derived from patients who underwent surgery at Nagoya University Hospital from April 2011 to December 2015 and who were diagnosed with lung adenocarcinoma, squamous cell carcinoma, or SCLC by pathologists. For the in vivo experiments, mixed tumors from mice were harvested, and 4‐μm thick formalin‐fixed, paraffin‐embedded sections were prepared. A Histofine HER2 Kit MONO (Nichirei Biosciences, Inc., Tokyo, Japan) was used to detect the HER2 protein, according to manufacturer instructions. HER2 expression was evaluated according to the guidelines for HER2 staining in breast cancer tissues.

### 5.8 Flow cytometry

Cells (1 × 10^5^) were seeded and incubated with Tra–IR700 (10 μg/ml) or T‐DM1–IR700 (10 μg/ml) for 6 h at 37°C, and the fluorescence intensity of IR700 was measured in 10,000 cells on a flow cytometer (Gallios; Beckman Coulter, Brea, CA, USA). A blocking study was performed to demonstrate the specific binding between HER2 and Tra–IR700 or T‐DM1–IR700. The cells were incubated with excess unconjugated Tra (100 μg) or T‐DM1 (100 μg) for 6 h at 37°C to saturate HER2 receptor binding, followed by the addition of Tra–IR700 (10 μg) or T‐DM1–IR700 (10 μg), respectively.

### 5.9 In vitro cell‐growth assay

Cells (5 × 10^4^) were seeded in 24‐well plates. After an overnight incubation, serially diluted S‐Me‐DM1 or mAb–IR700 complex was added to the wells, and the cells were incubated for 4 days. Cell viabilities were evaluated based on luciferase activities, which were determined using a plate reader (Powerscan 4; BioTek, Winooski, VT, USA) at 4 days after. For the luciferase assay, 200 μl of 150 μg/ml d‐luciferin‐containing media (GoldBio, St Louis, MO, USA) was added to the cells after they were washed with phosphate‐buffered saline (PBS).

### 5.10 Fluorescence microscopy

Cells (1 × 10^4^) were seeded in 35‐mm glass‐bottomed dishes and incubated overnight at 37°C with Tra–IR700 (10 μg/ml) or T‐DM1–IR700 (10 μg/ml). After washing with PBS, PI (diluted 1:2000; Thermo Fisher Scientific) was added and the cells were incubated for 30 min to stain for dead cells. Cells were then irradiated with NIR light (4 J/cm^2^) and observed under a fluorescence microscope (TiE‐A1R; Nikon Instech, Tokyo, Japan).

### 5.11 In vitro NIR‐PIT

Cells (1 × 10^5^) were seeded in 12‐well dishes and incubated with Tra–IR700 (10 μg/ml) or T‐DM1–IR700 (10 μg/ml) for 6 h at 37°C. For NIR‐PIT, cells were irradiated with 4 J/cm^2^ of NIR light from a 690 nm‐Laser (MLL‐III‐690, Changchun New Industries Optoelectronics Tech, CO., Ltd, Changchun, China). The actual power density (mW/cm^2^) was determined using an optical power meter (PM100; Thorlabs, Newton, NJ, USA), as previously reported.^42^ Cell viability was evaluated based on luciferase activity.

For the luciferase assay for in vitro mixed culture, 200 μl of 150 μg/ml d‐luciferin‐containing media was added to PBS‐washed cells.

For evaluating NIR‐PIT in vitro on the single cell line (3T3/HER2 or MDAMB468‐luc) in Figure 3c, after washing twice with PBS, cells were irradiated with 4 J/cm^2^ of NIR light and at 1 h later, cell viability was evaluated.

For evaluating NIR‐PIT in vitro on the mixed cell lines (Figure 3d and Figure S8), after washing twice with PBS, replacing phenol red free media 300 μl, then cells were irradiated with 4 J/cm^2^ of NIR light. At 4 days after NIR‐PIT, the viability of luciferase‐expressing cells was evaluated using a bioluminescence plate reader (Powerscan 4).

### 5.12 In vivo NIR‐PIT

Tra–IR700 (3.6 μg/g) or T‐DM1–IR700 (3.6 μg/g) was administered intravenously to mice on Day −1 (6 days after tumor cell transplantation). The dose similar to that of T‐DM1 administered to humans (3.6 mg/kg). The NIR‐light was irradiated at 1 and 2 days after the drug administration (Figure 4d).^43^, ^44^ The tumors on mice were then irradiated with NIR light (15 J/cm^2^ on Day 0 and 30 J/cm^2^ on Day 1) with a 690 nm laser. The antitumor effects of NIR‐PIT were evaluated based on luciferase activity, estimated tumor volume, and duration of survival.

### 5.13 In vivo FLI

Tra–IR700 was administered intravenously to mice, and the IR700 FLI was assessed using a Pearl Imager (LI‐COR Biosciences).^45^

### 5.14 In vivo BLI

For BLI, d‐luciferin (7.5 mg/ml, 200 μl) was administered intraperitoneally to mice, and luminescence images were obtained 10 min later using an IVIS imaging system (PerkinElmer).^46^ The luciferase activity was evaluated as the average radiance (p·s^−1^·cm^−2^/r^−1^), using Living Image Software (Perkin Elmer).

### 5.15 Preparation for the in vitro MS samples

For detecting released DM1 derivatives in tube, the T‐DM1‐IR700 (500 μg/ml) in PBS was irradiated with a 690 nm NIR laser. The sample was centrifuged with 10 K membrane Amicon Ultra (Merk, Darmstadt, Germany) to remove mAb‐derived proteins, and the 10 K passed solution was analyzed with the MS.

For detecting released DM1 derivatives in vitro mixed culture, the 10 μg/ml T‐DM1‐IR700 in PBS was incubated with the mixed cells for 6 h. After washing cells twice with PBS, cells were NIR‐light irradiated. The supernatant of the mixed cell culture was collected, then the sample was centrifuged with 10 K membrane Amicon Ultra to remove proteins, and the 10 K passed solution was analyzed with the MS.

### 5.16 Mass spectroscopy

Liquid chromatography‐tandem MS (LC–MS/MS) was conducted using a QTRAP6500 system (AB Sciex, Framingham, MA, USA) coupled to a Shimadzu Prominence LC system (Shimadzu Co., Kyoto, Japan). For LC separation, we used an L‐column 2 ODS semi‐micro column (150 mm × 1.5 mm i.d.; pore size, 120 Å; 3 μm particles; Chemicals Evaluation and Research Institute, Tokyo, Japan). Analytes were chromatographically separated using linear‐gradient elution with mobile phases A (0.1% formic acid and 5% acetonitrile) and B (0.1% formic acid and 0.1% acetonitrile) at a flow rate of 0.1 ml/min (0–15 min, B: 0%–100%). The column oven was maintained at 40°C, and the injection volume was set at 5 μl. LC–MS/MS was performed in the multiple reaction monitoring (MRM) mode. The MRM transitions and other MS parameters were as follows: MPM transition precursor ion, 752.159 mass: charge (m/z) ratio; product ion, 485.100 m/z ratio; time, 50 ms; declustering potential, 81 V; entrance potential; 10 V; collision energy, 31 V; and collision cell exit potential, 38 V.

### 5.17 Quantification and statistical analysis; statistical analysis

Data are expressed as the mean ± SEM of a minimum of three experiments, unless otherwise indicated. Statistical analyses were conducted using Prism software (GraphPad Software, San Diego, CA, USA). For two‐group comparisons, a Student's unpaired *t*‐test was used. For multiple‐group comparisons, one‐way analysis of variance with Tukey's test or Dunnett's test was used. The cumulative probability of survival, defined as the nonachievement of a tumor diameter of 20 mm, was estimated in each group using Kaplan–Meier analysis, and the results were compared using the log‐rank and Wilcoxon tests. *p* < 0.05 was considered to reflect a statistically significant difference.

## AUTHOR CONTRIBUTIONS

**Kazuomi Takahashi:** Data curation (lead); formal analysis (lead); investigation (lead); methodology (equal); validation (equal); visualization (equal); writing – original draft (equal); writing – review and editing (equal). **Hirotoshi Yasui:** Data curation (supporting); formal analysis (supporting); investigation (supporting); methodology (supporting); resources (supporting); software (supporting); validation (supporting). **Shunichi Taki:** Data curation (supporting); formal analysis (supporting); investigation (supporting); software (supporting); validation (supporting); visualization (supporting). **Misae Shimizu:** Formal analysis (supporting); methodology (supporting); resources (supporting); software (supporting); validation (supporting). **Chiaki Koike:** Data curation (supporting); methodology (supporting); resources (equal); software (supporting); visualization (supporting). **Kentaro Taki:** Data curation (equal); formal analysis (equal); investigation (equal); methodology (equal); software (equal). **Hiroshi Yukawa:** Formal analysis (supporting); resources (supporting); software (supporting). **Yoshinobu Baba:** Resources (supporting); software (supporting). **Hisataka Kobayashi:** Methodology (equal); validation (equal). **Kazuhide Sato:** Conceptualization (lead); data curation (lead); formal analysis (lead); funding acquisition (lead); investigation (lead); methodology (equal); project administration (lead); resources (lead); software (equal); supervision (lead); validation (lead); visualization (lead); writing – original draft (lead); writing – review and editing (lead).

## CONFLICT OF INTEREST

Kazuhide Sato and Kazuomi Takahashi have a patent pending. The other authors declare no conflicts of interest.

### PEER REVIEW

The peer review history for this article is available at https://publons.com/publon/10.1002/btm2.10388.

## Supporting information


**Appendix S1** Supporting Information

## Data Availability

Data and materials are available upon reasonable request. All data relevant to the study are included in the article or are uploaded as supplementary information.
