# Supplementary material for: Near‐infrared‐induced drug release from antibody–drug double conjugates exerts a cytotoxic photo‐bystander effect
Source: Bioeng Transl Med. 2022 Aug 21;7(3):e10388. doi: 10.1002/btm2.10388 (PMC9471993; doi:10.1002/btm2.10388)
Supplement: Supplementary file 1 — Appendix S1 Supporting Information [file BTM2-7-e10388-s001.pdf]

Supplementary Information

Near-infrared-induced drug release from antibody–drug double conjugates exerts a cytotoxic photo-bystander effect

Kazuomi Takahashi[1†], Hirotoshi Yasui[1], Shunichi Taki[1], Misae Shimizu[2], Chiaki Koike[2], Kentaro Taki[3], Hiroshi Yukawa[2,4,5], Yoshinobu Baba[4,5], Hisataka Kobayashi [6], Kazuhide Sato[1,2,4,7,8 † *]

[1] Respiratory Medicine, Nagoya University Graduate School of Medicine
[2] Nagoya University Institute for Advanced Research, Advanced Analytical and Diagnostic Imaging Center (AADIC) / Medical Engineering Unit (MEU), B3 Unit
[3] Division for Medical Research Engineering, Nagoya University Graduate School of Medicine
[4] Nagoya University Institute of Nano-Life-Systems, Institutes of Innovation for Future Society
[5] Department of Biomolecular Engineering, Nagoya University Graduate School of Engineering
[6] Molecular Imaging Program, National Cancer Insitute, National Institutes of Health, USA
[7] FOREST-Souhatsu, CREST, JST
[8] Nagoya University Institute for Advanced Research, S-YLC
[†] These authors are equally contributed to this work

***Correspondence should be addressed to:** Kazuhide Sato, M.D., Ph.D.
Institute for Advanced Research, Department of Respiratory Medicine, Graduate School of Medicine, Nagoya University, Nagoya, Aichi, 466-8550, Japan
Phone: +81-052-744-2167; Fax: +81-052-744-2176;
E-mail: k-sato@med.nagoya-u.ac.jp

Figure S1

Sample	m/z (observed)	Intensity	Relative	Charge	Formula
S-Me-DM1	752.2944	84588344	100.00	1	[M+H]
16J	752.2609	13975.8	40.90	1	[M+H]
S-Me-DM1	734.2844	36042688	42.61	1	[M+H-H2O]
S-Me-DM1	690.2956	41710800	49.31	1	[M+H-H2O-CO2]
16J	690.2545	4044.5	11.84	0	[M+H-H2O-CO2]
S-Me-DM1	547.2212	1542792.9	53.55	1	[M1]
S-Me-DM1	529.2082	5009398.5	5.92	1	[M1-H2O]
16J	529.2087	16618.5	3.86	0	[M1-H2O]
S-Me-DM1	485.2202	2117248.5	73.49	1	[M1-H2O-CO2]
16J	485.2202	34170.2	100.00	1	[M1-H2O-CO2]
S-Me-DM1	453.1939	2358421.5	81.86	1	[M1-H2O-CO2 -CH3OH]
16J	453.1940	15243.8	44.61	1	[M1-H2O-CO2 -CH3OH]
S-Me-DM1	435.1834	1115393.1	38.71	1	[M1-H2O-CO2 -CH3OH-H2O]
16J	435.1842	127534.4	29.62	1	[M1-H2O-CO2 -CH3OH-H2O]
S-Me-DM1	425.1988	2712884.3	3.21	0	[M1-H2O-CO2 -CH3OH-CO2]
16J	425.2016	23892.4	5.55	0	[M1-H2O-CO2 -CH3OH-CO2]
S-Me-DM1	188.0740	2881042.5	100.00	1	[M2]
16J	188.0377	17258.7	4.01	0	[M2]
S-Me-DM1	160.0790	1650490.4	57.29	0	[M2-CO]
16J	160.0430	2740.6	0.64	0	[M2-CO]

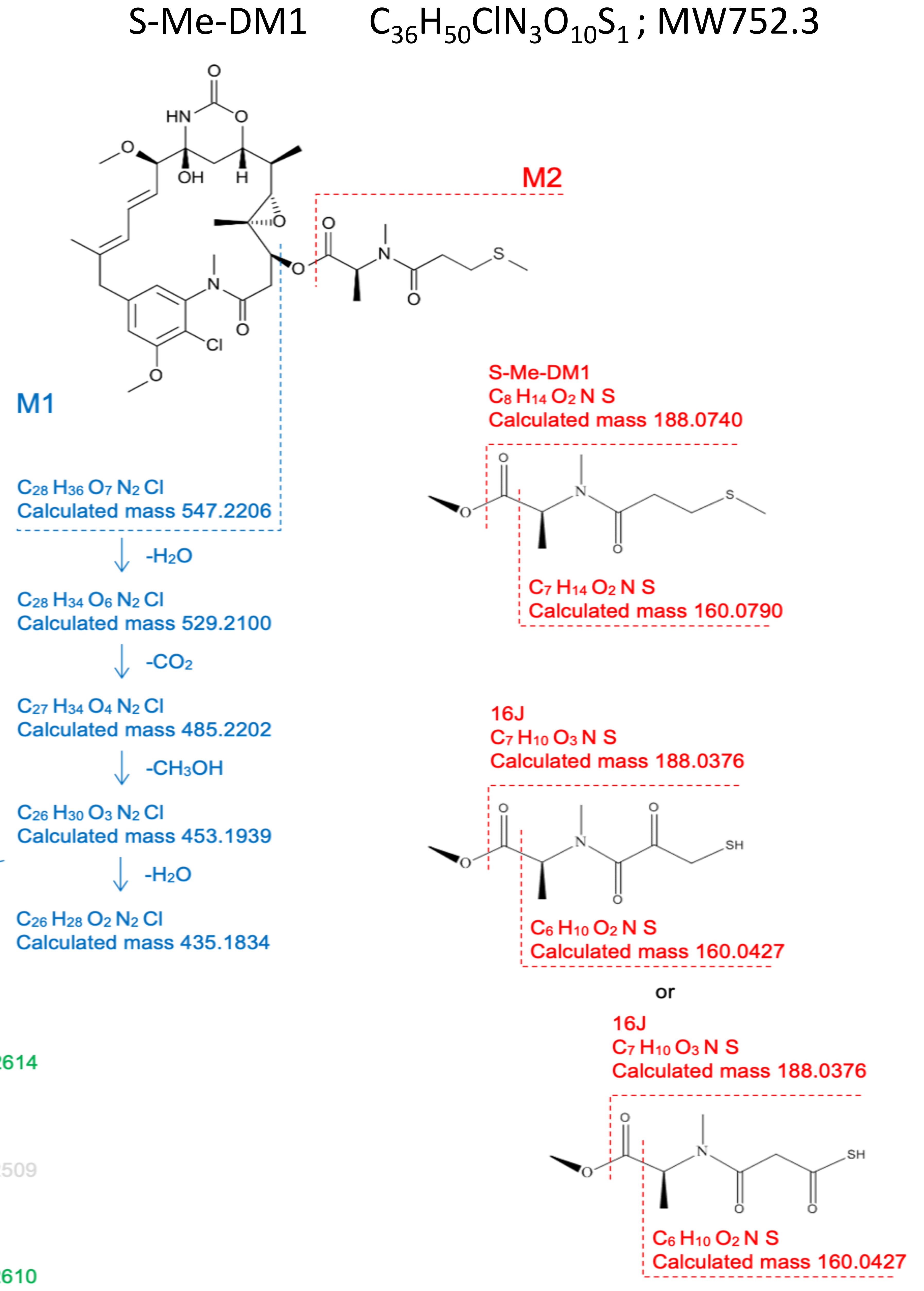

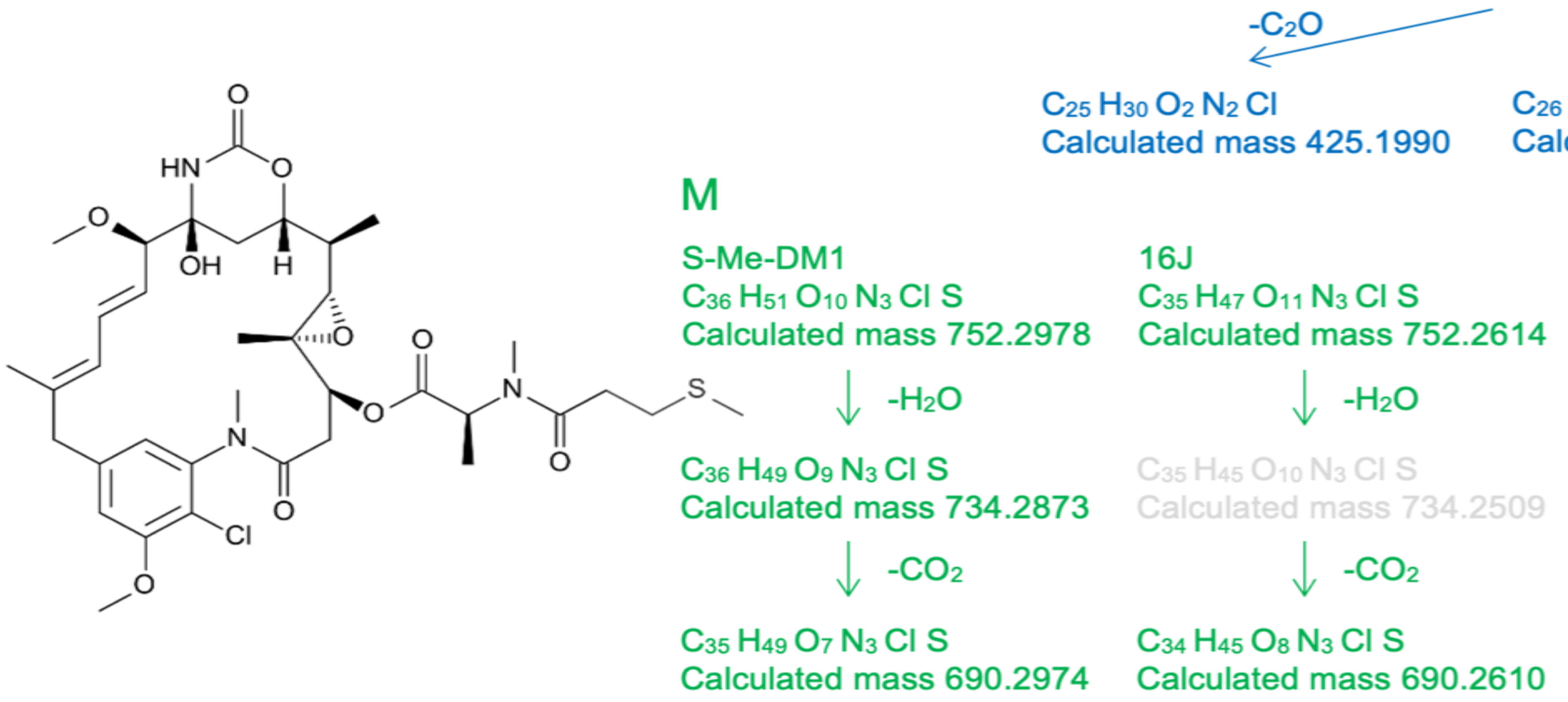

Fig. S1.

Derivatives of T-DM1-IR700 obtained by NIR-light irradiation were further confirmed by product ion analysis using a high resolution mass spectrometer.

Further fragmentation with high resolution mass spectrometer confirmed the detected peak as $C_{36}H_{50}ClN_3O_{10}S_1$.

Figure S2

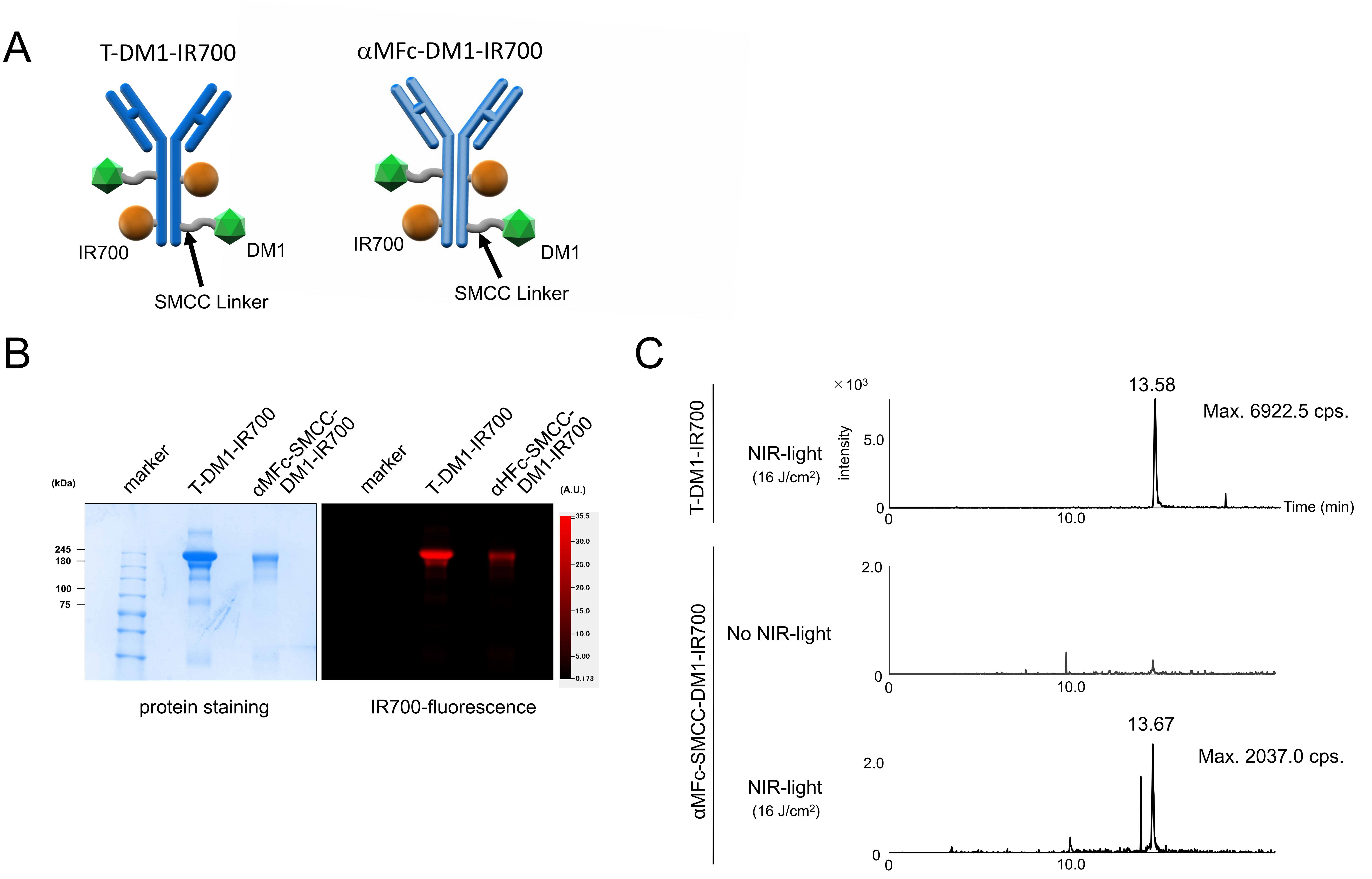

Fig. S2.

Changing the antibody from T-DM1-IR700 (αMFc-DM1-IR700) could be photo-released DM1.

A, Scheme of the T-DM1-IR700 and αMFc-DM1-IR700 is shown here. The core antibody is changed to αMFc from trastuzumab. **B,** SDS-PAGE showed the successful conjugation of IR700 to the antibody (left: colloidal blue protein staining, right: fluorescence image at 700 nm). **C,** The analysis of NIR-light irradiated T-DM1-IR700 and aMFc-DM1-IR700 using LC-MS/MS showed that each peak has similar retention time, suggesting that they were DM-1 derivatives.

Figure S3

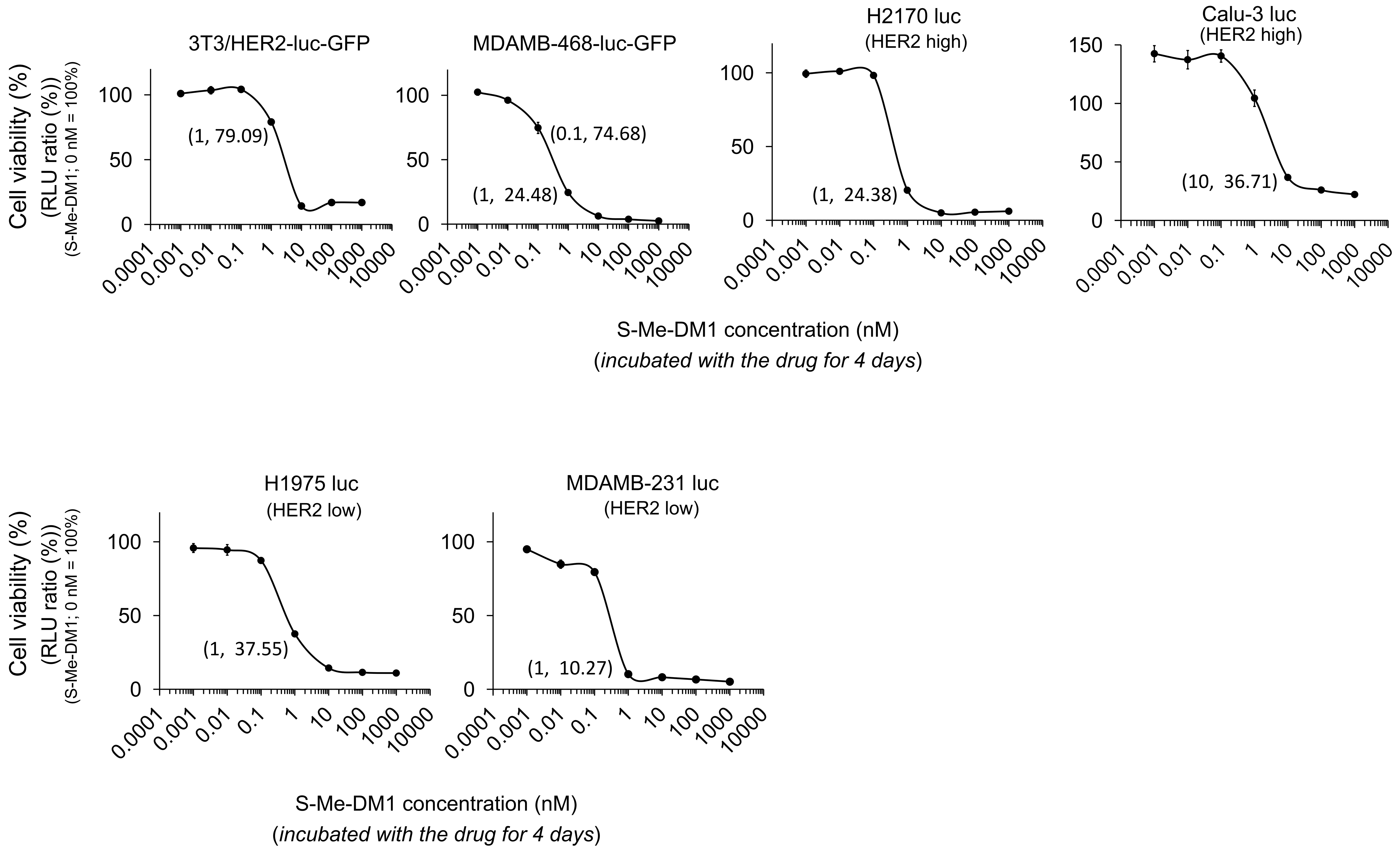

Fig. S3.

Inhibitory concentration with S-Me-DM1 was tested on 3T3/HER2 (high HER2 expression), MDAMB-468 (negative HER2 expression), H2170 (high HER2 expression), Calu3 (high HER2 expression), H1975 (low HER2 expression), and MDAMB-231 (negative HER2 expression).

The cytotoxicity of S-Me-DM1 for the various cell lines indicated was tested. S-Me-DM1 was added onto the media at the indicated concentration and incubated for four days. After four days of incubation with the drug, the cell viability was evaluated with luciferase assay. The ratios were calculated as 100 % at 0 nM. The inhibitory concentration of 50 % cell viability (IC50) ranged from 0.1 ~ 10 nM in various cell lines. Data are presented as means ± SEM (*n* = 4).

Figure S4

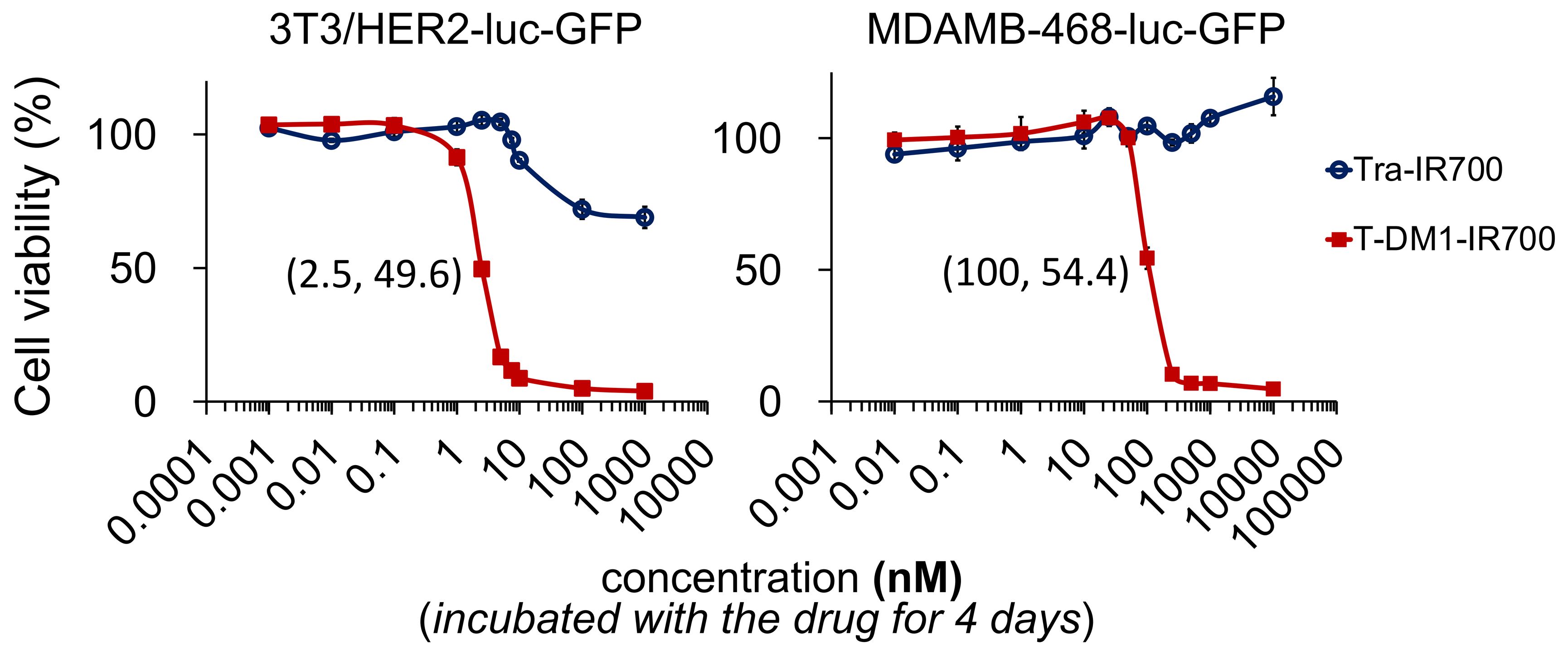

Fig. S4.

Inhibitory concentration with Tra-IR700 or T-DM1-IR700 was tested on 3T3/HER2 (high HER2 expression), and MDAMB-468 (negative HER2 expression.

Toxicity of Tra–IR700 and T-DM1–IR700 toward 3T3/HER2-luc-GFP and MDAMB-468-luc-GFP cells. Tra–IR700 or T-DM1–IR700 was added to the media at the indicated concentrations and incubated for 4 days, after which cell viabilities were evaluated by determining the luciferase activities. Luminescence ratios were calculated by setting the luminescence observed at 0 nM as 100%. The IC_{50} was ~40-fold higher for HER2− MDAMB-468-luc-GFP cells than for HER2+ 3T3/HER2-luc-GFP cells. Tra–IR700 inhibited the growth of 3T3/HER2-luc-GFP cells, but not that of MDAMB-468-luc-GFP cells (*n* = 4).

Figure S5

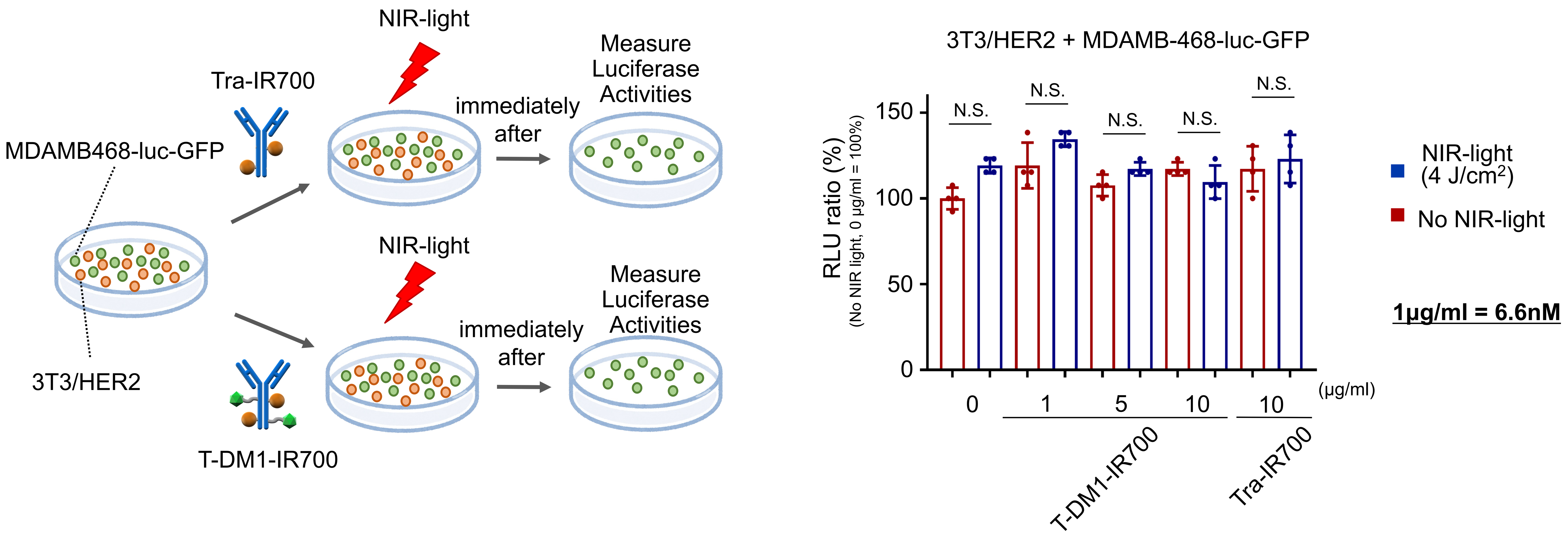

Fig. S5.

No *in vitro* significant effects on non-targeting cells in mixed-culture was detected immediately after Tra-IR700 or T-DM1-IR700 mediated NIR-PIT

3T3/HER2 and MDAMB-468-luc-GFP were co-mixed-cultured. NIR-PIT was performed using T-DM1-IR700 (1, 5, or 10 µg/ml) or Tra-IR700 (10 µg/ml). Luciferase activity as Relative Light Unit (RLU), which means non-targeted MDAMB-468-luc-GFP cell viability, was measured at immediately after NIR-light-irradiation (left panel). No significant cytotoxic effect on MDAMB-468-luc-GFP cells in the mixed-culture was detected at immediately after NIR-light-irradiation (Data are presented as means ± SD. n = 4, Student's t-test).

Figure S6

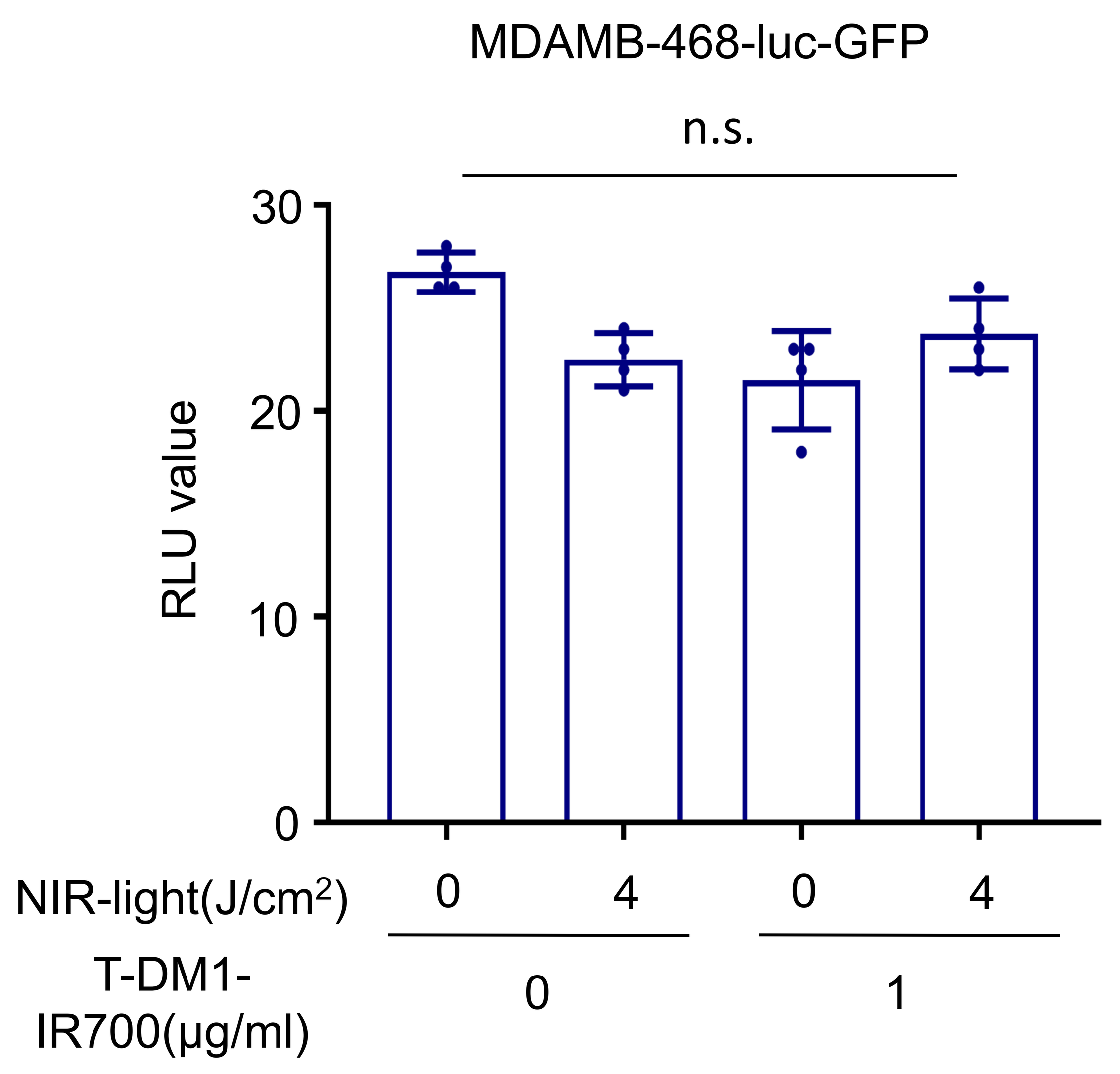

Fig. S6.
No effects of the NIR-PIT with T-DM1-IR700 against HER2-negative MDAMB-468-luc-GFP cells at Day-4

NIR-PIT with T-DM1-IR700 (1 μg/ml) against HER2-negative MDAMB-468-luc-GFP cells was performed, and the cells were incubated for four days with no changes of the irradiated medium (Data are presented as means ± SD. *n* = 4, Kruskal-Wallis test with Dunn's post-test).

Figure S7

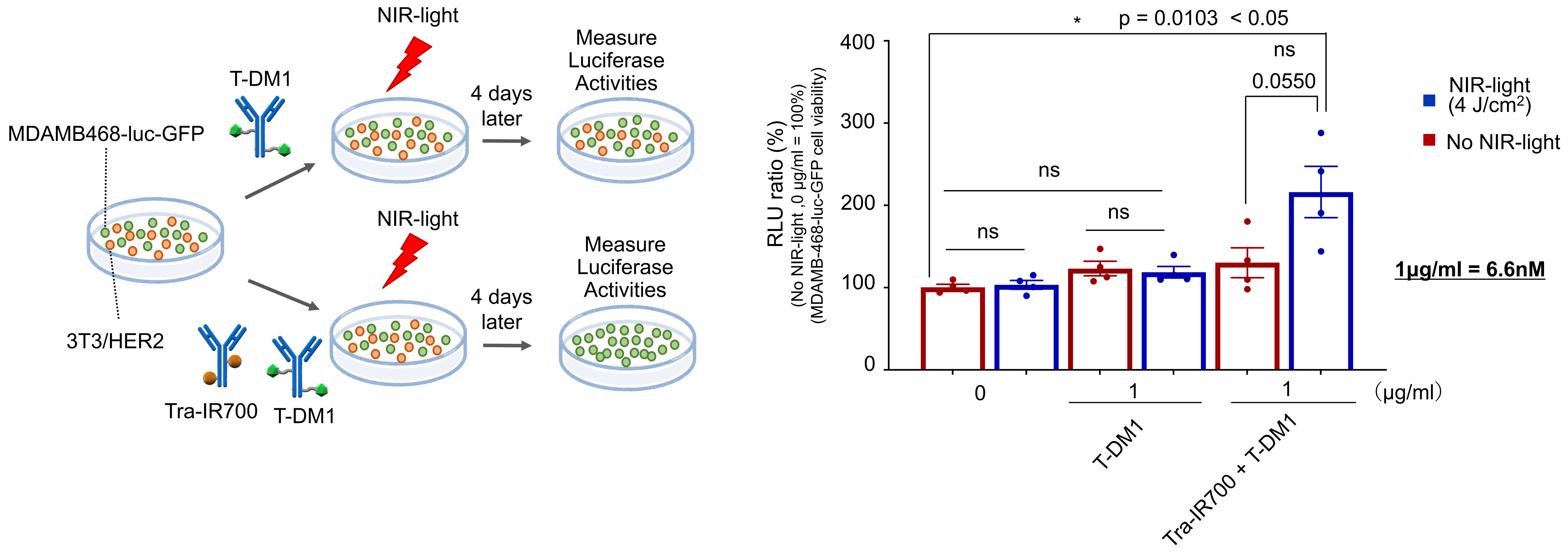

Fig. S7.

No *in vitro* significant effects on non-targeting cells (MDAMB-469-luc-GFP) in mixed-culture was detected with T-DM1 incubation, and a mixture of Tra-IR700 and T-DM1 with NIR-light grew non-targeting cells more than that without NIR-light or control.

3T3/HER2 and MDAMB-468-luc-GFP were co-mixed-cultured. They were incubated with T-DM1 (1 μg/ml) or a mixture of Tra-IR700 (1 μg/ml) and T-DM1 (1 μg/ml), and then they were irradiated with NIR-light (4 J/cm^2). Luciferase activity as Relative Light Unit (RLU), which means non-targeted MDAMB-468-luc-GFP cell viability, was measured at immediately after NIR-light-irradiation (left panel). No significant cytotoxic effect on MDAMB-468-luc-GFP cells in the mixed-culture was detected with T-DM1 incubation either with or without NIR-light (Data are presented as means ± SD. n = 4, Student's t-test). Non-targeting cells (MDAMB-469-luc-GFP) in mixed-culture grew more in NIR-PIT with a mixture of Tra-IR700 and T-DM1 than control or that without NIR-light.

Figure S8

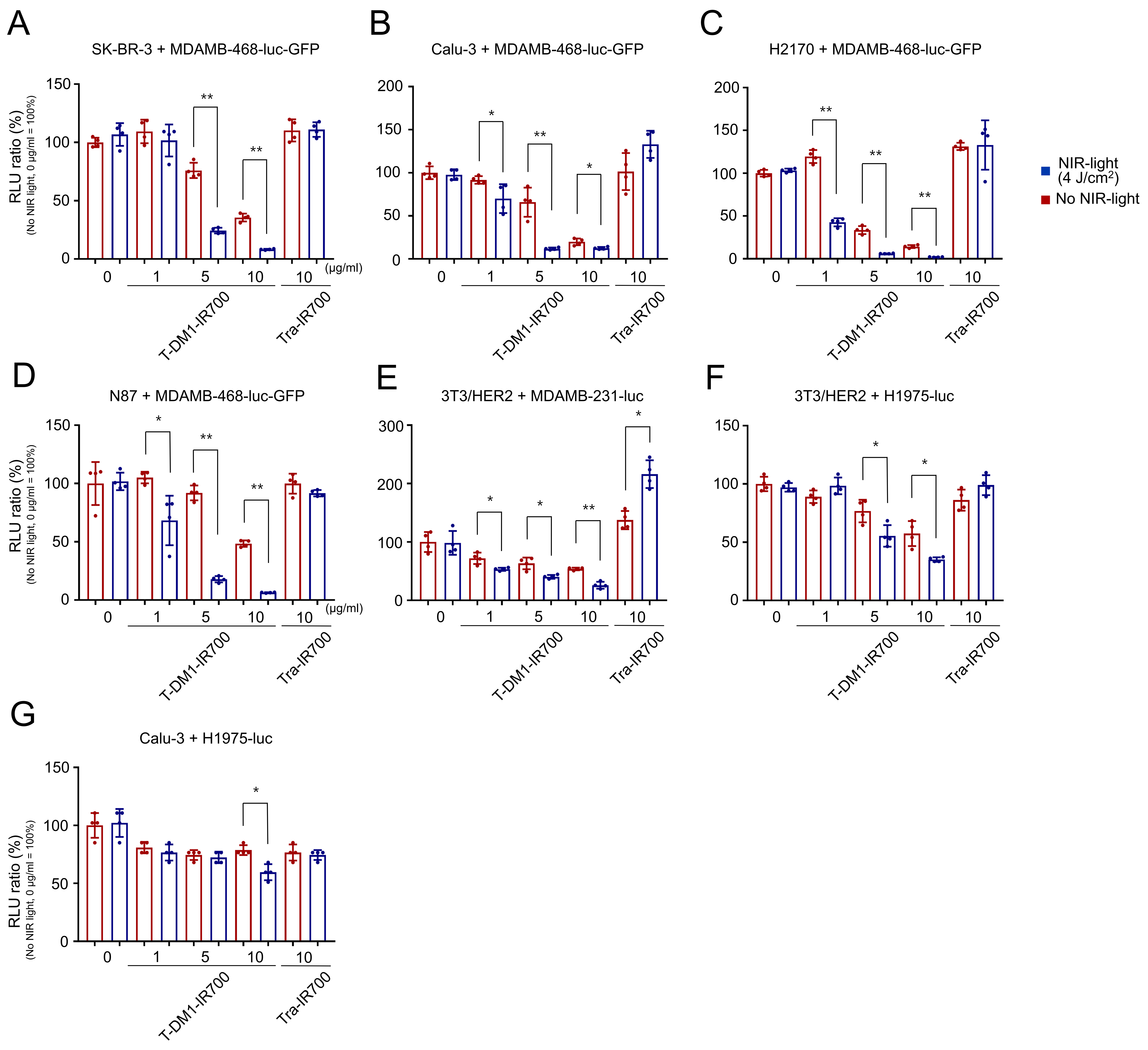
A
SK-BR-3 + MDAMB-468-luc-GFP
B
Calu-3 + MDAMB-468-luc-GFP
C
H2170 + MDAMB-468-luc-GFP
D
N87 + MDAMB-468-luc-GFP
E
3T3/HER2 + MDAMB-231-luc
F
3T3/HER2 + H1975-luc
G
Calu-3 + H1975-luc
RLU ratio (%)
(No NIR light, 0 μg/ml = 100%)
0
1
5
10
(μg/ml)
T-DM1-IR700
Tra-IR700
NIR-light (4 J/cm²)
No NIR-light

Fig. S8.

***In vitro* photo-bystander cytotoxic effect on various mixed-cultures.**

Various combinations of HER2 expressing cells were co-mixed-cultured are shown here. NIR-PIT was performed using T-DM1-IR700 (1, 5, or 10 µg/ml) or Tra-IR700 (10 µg/ml), and the mixed-culture was then incubated for 4 days. Luciferase activity represented as Relative Light Unit (RLU), that indicated the non-targeted or low-targeted cell-line viability, was measured four days after NIR-light irradiation (Data are presented as means ± SD. n = 4, *$p < 0.05$, **$p < 0.0001$, Student's t-test). Mixed co-culture of

A, SK-BR-3 (HER2-positive, breast cancer cell line) and MDAMB-468-luc-GFP (HER2-negative , breast cancer cell line),

B, Calu-3 (HER2-positive, lung adenocarcinoma cell line) and MDAMB-468-luc-GFP (HER2-negative),

C, H2170 (HER2-positive, lung squamous cell line) and MDAMB-468-luc-GFP (HER2-negative),

D, N87 (HER2-positive, gastric cancer cell line) and MDAMB-468-luc-GFP (HER2-negative),

E, 3T3/HER2 (HER2-positive) and MDAMB-231-luc (HER2-negative, breast cancer cell line),

F, 3T3/HER2 (HER2-positive) and H1975-luc (HER2-low),

G, Calu-3 (HER2-positive) and H1975-luc (HER2-low, lung adenocarcinoma cell line)

Figure S9

A

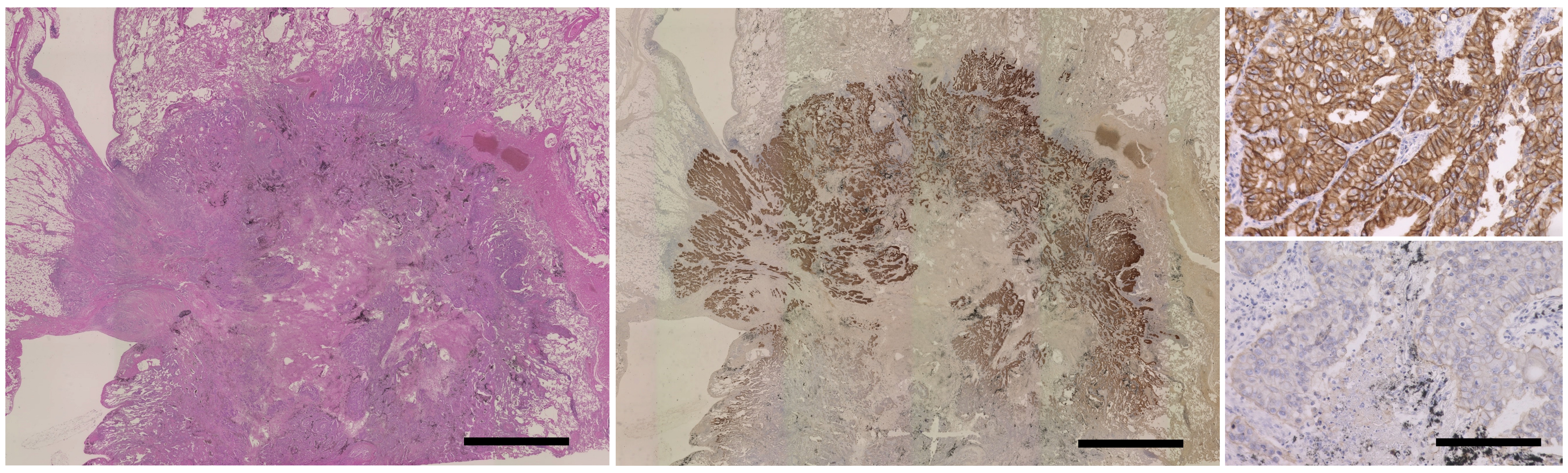

H-E stain HER2 immunohistochemistry

B

Histology	IHC score 0	1+	2+	3+	HER2 positive (2/3+) heterogeneity rate
NSCLC					
adeno (n=14)	7 (50%)	3 (21%)	3 (21%)	1 (8%)	4/4 (100%)
squamous (n=12)	11 (92%)	1 (8%)	0 (0%)	0 (0%)	0/0 (0%)
SCLC (n=5)	5 (100%)	0 (0%)	0 (0%)	0 (0%)	0/0 (0%)

Fig. S9.

Immunohistochemical staining of the resected surgical specimens of non-small cell lung cancer (NSCLC) and small cell lung cancer (SCLC)

A, A representative image of Hematoxylin-Eosin (HE) and HER2 staining in the resected NSCLC adenocarcinoma specimens. The tumor region was heterogeneously positive for HER2. Scale bars, whole tumor view = 5 mm. Magnified view of immune staining = 500 µm

B, The HER2-immunostained specimens have 100% heterogeneity. All the adeno NSCLC specimens have IHC scores in the range of 0+ to 3+ in the same tumor, indicating that the “HER2 positive” NSCLC adeno cancers show antigen heterogeneity. NSCLC squamous have 8 % at 1+ IHC score, and 92% was negative. All SCLC specimens were negative for HER2 immunostaining.

Figure S10

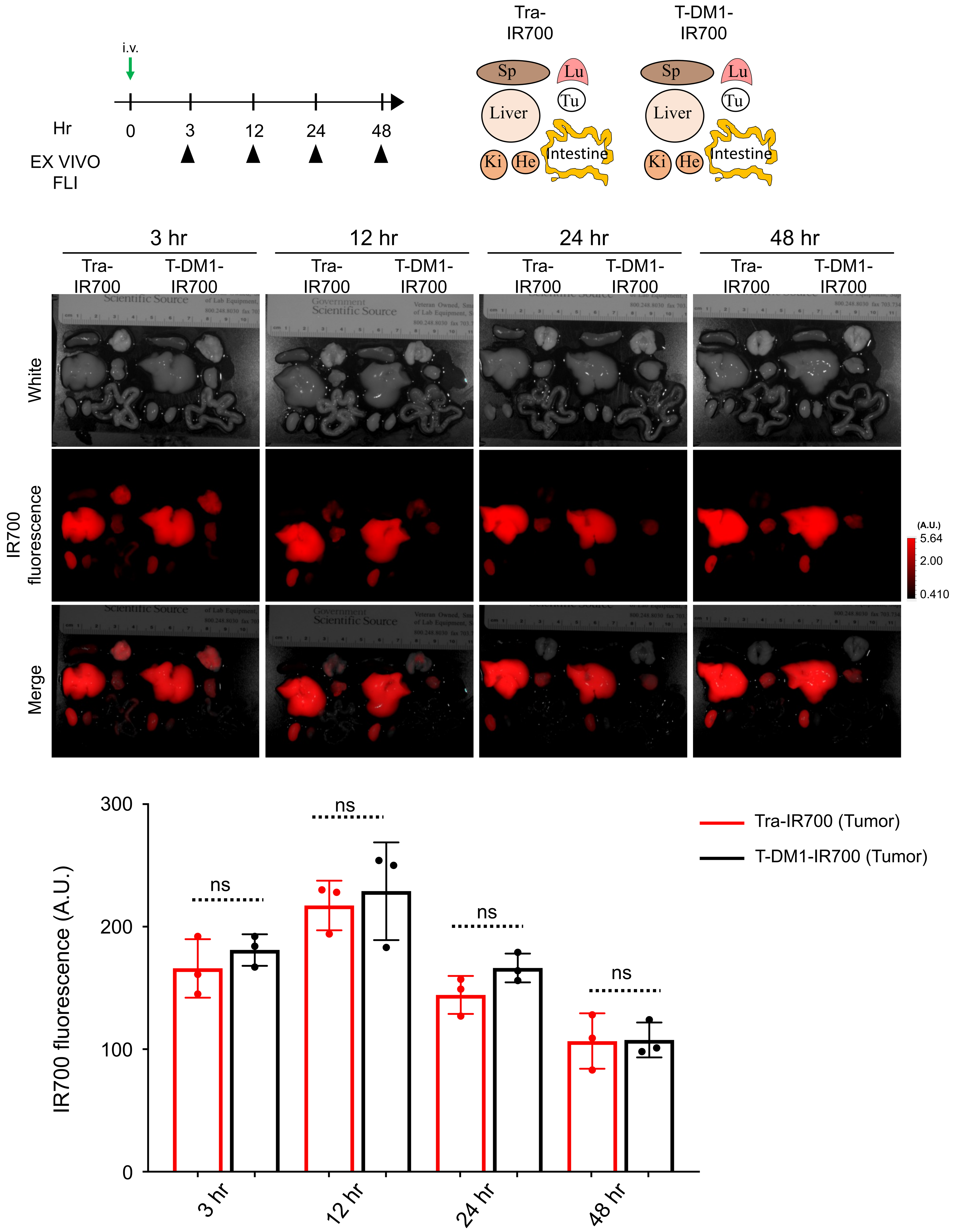

Fig. S10.

***Ex vivo* evaluation of distribution via IR700-fluorescence of Tra-IR700 or T-DM1-IR700 after intravenous administration.**

(A) Regimen of *ex vivo* evaluation was indicated. IR700-fluorescence images (FLI) were taken at the several time points. Left panel demonstrated the positions of organs.

(B) Representative IR700-FLIs at different time points after the administration were demonstrated. Sp: spleen, Lu: lung, Tu: tumor, Ki: kidney, He: heart

(C) Quantitative IR700-fluorescence on the tumors was compared between Tra-IR700 and T-DM1-IR700 at the different time points (n = 3, ns; not significant, unpaired student's t test).

Figure S11

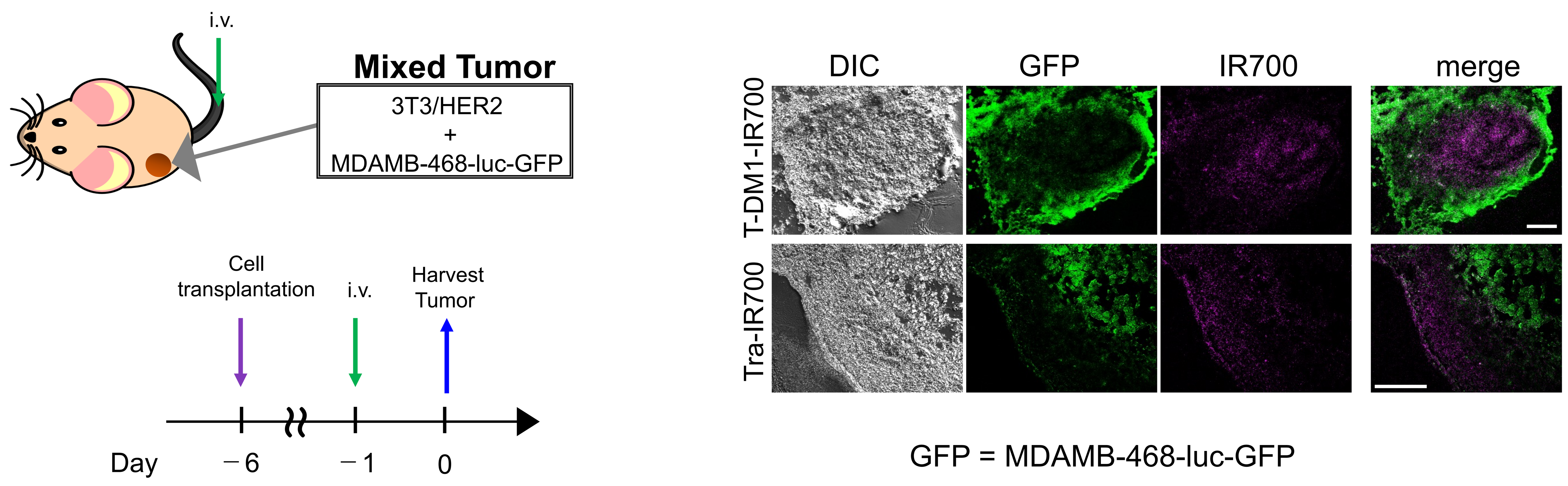

Fig. S11.
Colocalization of Tra-IR700 or T-DM1-IR700 with 3T3/HER2 tumor population in the mixed tumor *in vivo*.

At 5 days after the inoculation of the mixed cells of 3T3/HER2 and MDAMB-468-luc-GFP cells, Tra-IR700 or T-DM1-IR700 was injected intravenously via tail vein. At 1 day after the administration, the mixed tumor was harvested and frozen. The, the frozen tumors were sliced and observed with a fluorescence microscopy. GFP showed the population of MDAMB468-luc-GFP cells, where IR700 fluorescence less colocalized than non-GFP tumor regions. Bar = 1 mm

Figure S12

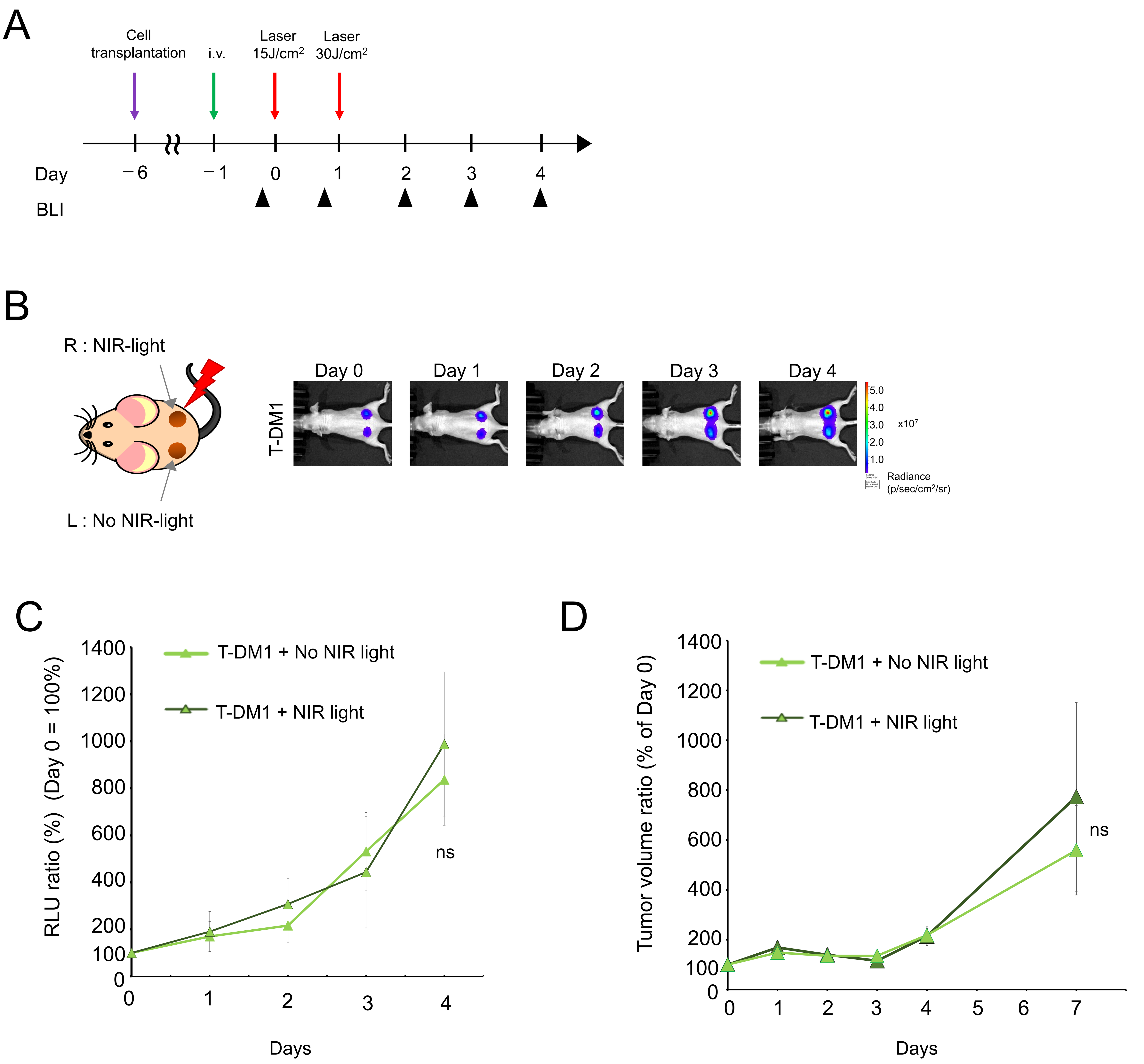

Fig. S12.

Evaluation of the *in vivo* cytotoxic bystander effect with T-DM1.

(A) *In vivo* therapeutic regimen involving tumor cell inoculation, T-DM1 injection, and NIR-light exposure. BLI was performed at the indicated points (arrowheads). BLI indicated HER2− non-targeted MDAMB-468-luc-GFP tumor activity in the mixed tumor.

(B) Mixed tumors inoculated on both dorsa of mice, with only the right-sided tumor irradiated with NIR light. Representative BLI with T-DM1 administration is shown.

(C) Quantitative RLUs, indicating non-targeted HER2− MDAMB-468-luc-GFP cells inside the mixed tumors (*n* = 4 mice/group). Gradual increase of luciferase activities of mixed tumors with T-DM1 administration was detected.

(D) Mixed tumor volume (mm^3) with T-DM1 administration (defined as day 0 = 100; *n* = 4 mice/group).

Figure S13

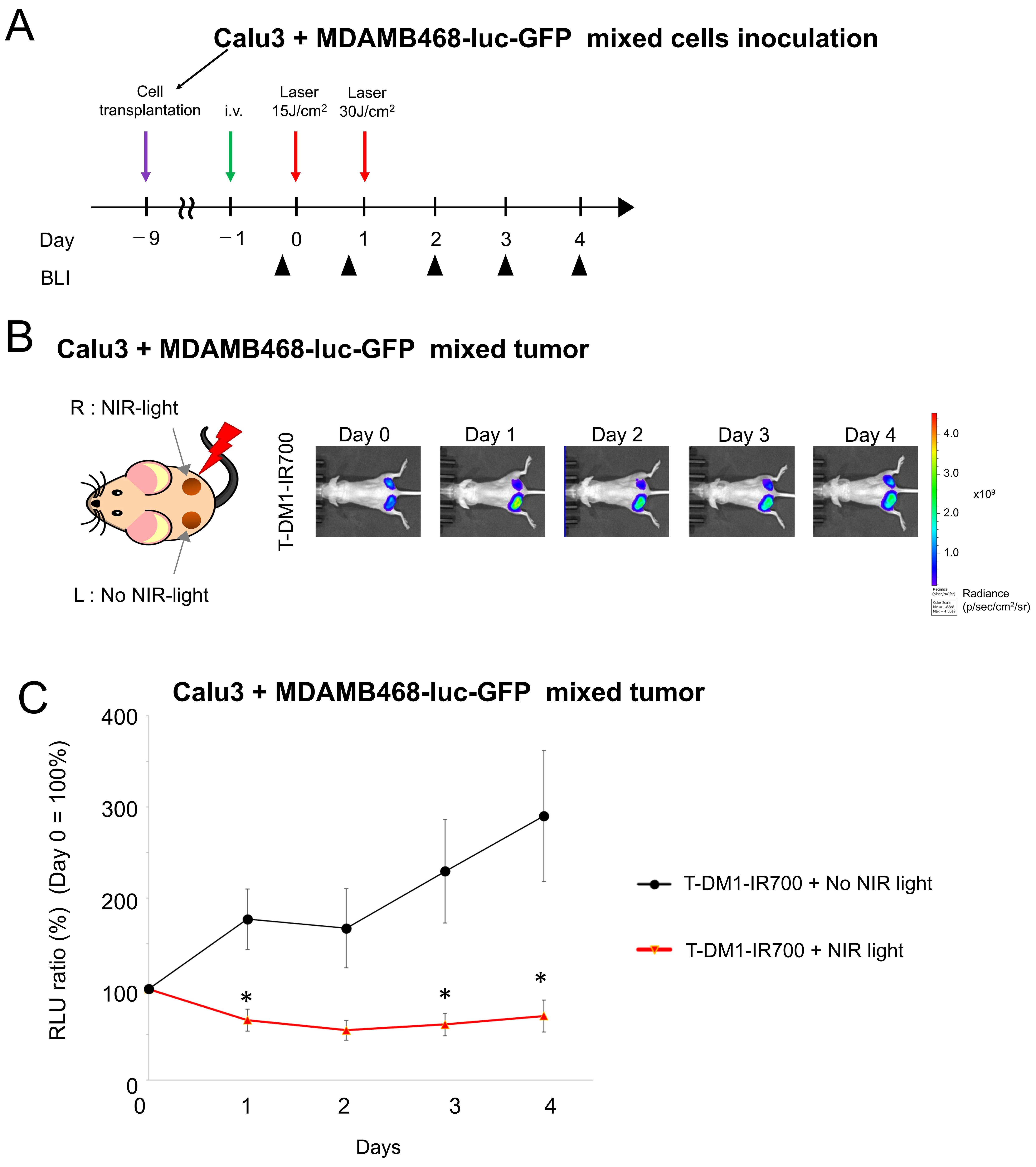

Fig. S13.

Evaluation of the *in vivo* cytotoxic bystander effect with T-DM1-IR700 on Calu3 and MDAMB-468-luc-GFP mixed tumor.

(A) *In vivo* therapeutic regimen involving tumor cell inoculation (Calu3 and MDAMB-468-luc-GFP mixed cells), T-DM1-IR700 injection, and NIR-light exposure. BLI was performed at the indicated points (arrowheads). BLI indicated HER2− non-targeted MDAMB-468-luc-GFP tumor activity in the mixed tumor.

(B) Mixed tumors inoculated on both dorsa of mice, with only the right-sided tumor irradiated with NIR light. Representative BLI is shown.

(C) Quantitative RLUs, indicating non-targeted HER2− MDAMB-468-luc-GFP cells inside the mixed tumors (*n* = 5 mice). Gradual increase of luciferase activities of mixed tumors without NIR-light was detected, while decrease of luciferase activities of mixed tumors with NIR-light was showed (* $p < 0.05$, unpaired t-test, n=5).
